# Notch2−expressing CD4^+^ T cells attain immunoregulatory functions during autoimmune inflammation

**DOI:** 10.1038/s41423-025-01318-2

**Published:** 2025-07-23

**Authors:** So-Eun Bae, Sang-Heon Park, Chae Youn Kim, Cho-Rong Lee, Chanyeon Lee, Rosah May Payumo, So Yeon Kim, Kyu-Young Sim, Ho Jin Kim, Hyungseok Seo, Seong-Joon Koh, Seunghee Hong, Sung-Gyoo Park

**Affiliations:** 1 Institute of Pharmaceutical Sciences, College of Pharmacy, Seoul National University, Seoul, Republic of Korea; 2 Department of Biochemistry, College of Life Science and Biotechnology, Yonsei University, Seoul, Republic of Korea; 3 National Cancer Center Graduate School of Cancer Science and Policy, Goyang, Republic of Korea; 4 Division of Clinical Research, Research Institute, National Cancer Center, Goyang, Republic of Korea; 5 Department of Neurology, Hospital of National Cancer Center, Goyang, Republic of Korea; 6 Division of Gastroenterology and Hepatology, Department of Internal Medicine, College of Medicine, Seoul National University, Seoul, Republic of Korea; 7 Brain Korea 21 (BK21) FOUR Program, Yonsei Education & Research Center for Biosystems, Yonsei University, Seoul, Republic of Korea

**Keywords:** Autoimmunity, CD4 T cell, Notch2, Dysfunction, Immune Regulation, Lymphocyte activation, Cell death and immune response

## Abstract

Autoantigen−specific CD4^+^ T cells are central to the development of autoimmune diseases, while the expansion of regulatory T (Treg) cells expressing Forkhead box protein 3 (Foxp3) is essential for mitigating these conditions. In this study, we identified CD4^+^Notch2^+^Foxp3^lo^ T cells in the spinal cords of mice with experimental autoimmune encephalomyelitis (EAE), dextran sodium sulfate−induced colitis model mice, and patients with ulcerative colitis as immune regulatory cells. These cells exhibited a nonproliferative, dysfunctional phenotype and demonstrated immune regulatory functions, including suppressive activity against activated CD4^+^ T cells and marked Treg cell expansion activity. Our data revealed that Notch2 deletion in Foxp3−expressing cells diminishes the ability of this population to reverse the clinical symptoms of EAE. Collectively, these findings suggest that Notch2 expression in dysfunctional CD4^+^ T cells plays a crucial role in immune regulation.

## Introduction

Approximately 3–5% of the global population suffers from at least 80 types of autoimmune diseases, including multiple sclerosis (MS) and inflammatory bowel disease (IBD) [1]. These conditions arise from abnormal immune responses; these responses are mediated by self−reactive immune cells, an inflammatory environment, and various other factors [1]. During autoimmune responses, T cells function as both immune activators and regulators. While CD4^+^ T helper (Th) cells protect against infection, certain subsets, such as CD4^+^IL−17a^+^ T cells, can trigger a decrease in barrier integrity, and autologous T cells attack self−tissues [2]; however, regulatory T (Treg) cells play a major role in regulating autoimmune disease [3]. Treg cells expressing Foxp3 regulate autoimmune induced inflammatory responses [3]. For example, Treg cells infiltrating the central nervous system (CNS) of patients with MS display reduced immune regulatory functions [4, 5]. Moreover, in a mouse model of experimental autoimmune encephalomyelitis (EAE), Treg cell numbers are increased during the remission phase [6–8], and several studies have shown that Treg cell transfer ameliorates symptoms of EAE [6, 9]. Additionally, Treg cells contribute to the complex pathogenesis of IBD, which includes Crohn’s disease and ulcerative colitis (UC), during onset or development [10]. The ability of transferred Treg cells to control inflammatory lesions has been demonstrated in several IBD models [11, 12]. Previous studies have demonstrated that Treg cells are closely associated with recovery from autoimmune disease and with amelioration of symptoms and that depletion of Treg cells in mice induces the onset of autoimmune disease [5, 13]. In addition, a recent study revealed that autoantigen−specific CD4^+^ T cells eventually develop an exhausted phenotype and express Foxp3. This study highlights that this population is common in several autoimmune diseases. However, studies have not clearly elucidated their exact role in inflammatory conditions [14].

Notch was identified over 110 years ago, and classic studies have shown that Notch signaling is an evolutionarily conserved pathway [15]. Notch signaling is a highly conserved pathway in metazoans. Mammals express four Notch receptors (Notch1−4) and five Notch ligands, namely, Delta−like (DLL) 1, 3, and 4 and Jagged (Jag) 1 and 2 [15, 16]. The diverse functions of Notch signaling are attributed to various combinations of Notch ligands and receptors [15]. After receptor–ligand binding, the Notch intracellular domain (NICD), which harbors a transcriptional activation domain, is released into the cytosol. The released NICD localizes to the nucleus and forms a complex with CBF1, a suppressor of hairlessness, Lag−1, a transcription factor family member [15, 16]. The complex subsequently regulates the expression of target genes. Notch signaling is involved in various biological phenomena, including neuronal functions [17], lineage commitment [18, 19], angiogenesis [20], and T−cell differentiation [21]. Thus, Notch signaling plays a crucial role in diseases, including T−cell lymphoblastic leukemia [22], Alagille syndrome [23], and MS [24]. Indeed, the Notch ligand expressed by antigen−presenting cells determines the fate of CD4^+^ helper T cells [25]. Additionally, the Notch signaling pathway is involved in the survival of memory CD4^+^ T cells, and Notch signaling promotes CD4^+^ T helper cell longevity [26]. Interestingly, the DLL4 and DLL1 Notch ligands aggravate EAE by inhibiting Treg cell differentiation and accumulation and increasing CNS inflammation [27, 28]. In contrast, Jag1, another Notch ligand, alleviates EAE, whereas blocking Jag1 expression with an anti−Jag1 antibody exacerbates EAE [28]. Although the mechanism is unknown, Notch signaling seems to be strongly correlated with autoimmunity [29].

Here, we show an association between Notch signaling and a dysfunctional phenotype of CD4^+^ T cells − similar to exhaustion and characterized by a nonproliferative state and expression of PD−1, Tim3, and Lag3 − in mouse models of MS (i.e., mice with EAE) and IBD (i.e., mice with dextran sodium sulfate (DSS)−induced colitis). We identified Notch2−expressing CD4^+^ T cells in the spinal cords of mice with EAE and the colons of colitis model mice and noted that these cells expressed low levels of Foxp3 in a manner dependent on the EAE phase. In addition, these cells are present in patients with IBD. Additionally, Notch2−expressing CD4^+^ T cells do not produce inflammatory cytokines; rather, they promote the differentiation and proliferation of Treg cells. Consistent with these in vitro results, we found that adoptive transfer of these cells alleviated EAE symptoms in mice, whereas Foxp3−expressing cell-specific Notch2-deficient mice experienced delayed remission. Thus, on the basis of our in vitro and in vivo results, we suggest that Notch2−expressing CD4^+^ T cells regulate immune responses and maintain the expression of Foxp3. These data provide novel insight into the role of Notch2 in the acquisition of immune modulatory functions in dysfunctional CD4^+^ T cells and suggest that this protein may be a therapeutic target for inflammatory diseases.

## Results

### Notch2−expressing CD4^+^ T cells are present in the spinal cords of mice with EAE

**Fig. 1:**
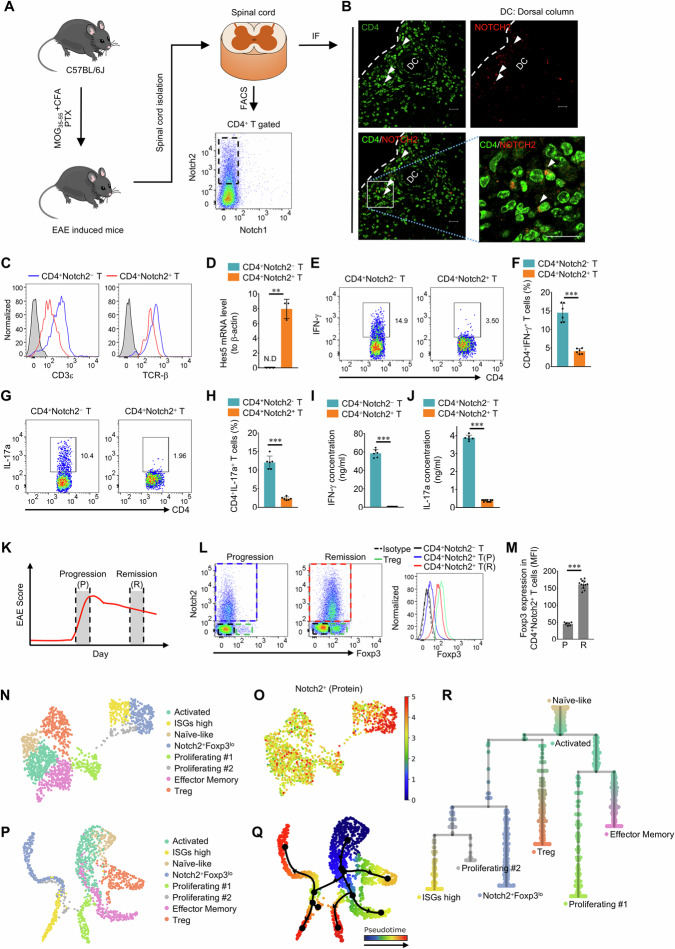
Notch2−expressing CD4^+^ T cells are present in the spinal cords of EAE mice. Spinal cords were harvested 10 −12 (P) or 21−25 (R) days after the induction of EAE. **A** Representative flow cytometry analysis of Notch1 and Notch2 expression in CD4^+^ T cells infiltrating the spinal cords of EAE mice. **B** Representative immunofluorescence analysis of CD4 (green) and Notch2 (red) expression in the spinal cords of EAE mice. Scale bar: 20 μm. **C** Representative flow cytometry analysis of CD3ε and TCR−β expression by CD4^+^ T cells infiltrating the spinal cords of EAE mice. **D** Representative qRT−PCR analysis of Hes5 mRNA expression in different spinal cord−infiltrating CD4^+^ T−cell types. The graph shows the means ± standard deviations (SDs) of Hes5 mRNA relative expression levels. **E** Representative flow cytometry analysis of IFN−γ^+^ cells within the spinal cord−infiltrating CD4^+^ T−cell population in EAE model mice. **F** Quantification of the frequency of CD4^+^IFN–γ^+^ T cells in the spinal cords of EAE mice. The graph shows the means ± SDs of the percentages of CD4^+^ IFN−γ^+^ T cells. **G** Representative flow cytometry analysis of IL−17a^+^ cells within the spinal cord−infiltrating the CD4^+^ T−cell population in EAE model mice. **H** Quantification of the CD4^+^IL−17a^+^ T−cell frequency in the spinal cords of EAE mice. The graph shows the means ± SDs of the percentage of CD4^+^IL−17a^+^ T cells. **I** Quantification of the cytokine bead array (CBA) results showing IFN−γ production by spinal cord−infiltrating CD4^+^ T cells after treatment with PMA/ionomycin. The graph shows the means ± SDs of IFN−γ secretion. **J** Quantification of the cytokine bead array (CBA) results showing IL−17a production by spinal cord−infiltrating CD4^+^ T cells after treatment with PMA/ionomycin. The graph shows the means ± SDs of IL−17a secretion. **K** P = progression of EAE symptoms, R = remission from EAE symptoms. **L** Representative flow cytometry analysis of Notch2 and Foxp3 expression, based on the mean fluorescence intensity (MFI). **M** Quantification of the MFI of Foxp3 in CD4^+^Notch2^+^ T cells in the progression and remission phases. **N** UMAP plot showing eight distinct CD4^+^ T−cell subclusters: activated, ISGs high, naïve−like, Notch2^+^Foxp3^lo^, proliferating #1, proliferating #2, effector memory and regulatory T (Treg) cells. **O** UMAP visualization of Notch2 protein expression across CD4^+^ T−cell populations. The color intensity indicates normalized protein expression levels. **P**. Re−calculated UMAP embedding for CD4^+^ T−cell trajectory analysis. **Q** Trajectory analysis of CD4^+^ T cells, with a Naïve−like cluster as the origin of differentiation. **R** Dendrogram summarizing developmental trajectories, starting from the naïve−like cluster and terminating in various CD4^+^ T−cell subclusters. The graph shows the mean ± SD of the Foxp3 MFI value. EAE, experimental autoimmune encephalomyelitis. N.D. Not−detected. The data are representative of three (**A**–**M**) independent experiments (error bars, s.d. of three (**D**) or > six (**F**, **H**–**J**, **M**) mice). Significance was determined by two−way ANOVA followed by an unpaired t−test in all the experiments; **P < 0.01, ***P < 0.001

In our examination of Notch1 and Notch2 expression in CD4^+^ T cells from the mice with EAE, we observed a trace amount of Notch1 and Notch2 expression in peripheral lymphoid organs (Supplementary Fig. 1A, B); however, while Notch2−expressing CD4^+^ T cells were readily detected in the spinal cord, Notch1−expressing CD4^+^ T cells were not observed (Fig. 1A). Notch3 and Notch4 were also analyzed in CD4^+^ T cells, but we did not observe their protein expression in most CD4^+^ T cells from the inguinal lymph nodes, spleen, or spinal cord of EAE mice (Supplementary Fig. 1C, D). This result likely indicates that Notch3 and Notch4 do not affect the majority of infiltrating CD4^+^ T cells in the inflamed spinal cord, although a minor population of CD4^+^ T cells may be influenced by Notch3 or Notch4. Immunofluorescence microscopy revealed that the colocalization of Notch2 with CD4 indicates that Notch2 is predominantly expressed on infiltrating CD4^+^ T cells during CNS inflammation. Moreover, these cells exhibited a scattered infiltration pattern within the spinal cord parenchyma rather than localizing to a specific region (Fig. 1B). Additionally, we confirmed that the Notch2−expressing CD4^+^ cells found in the CNSs of EAE mice were indeed T cells by identifying the presence of CD3ε and T−cell receptor (TCR) β expression on their surface, as these cells have not previously been reported in this context (Fig. 1C). We also investigated whether Notch2−expressing CD4^+^ T cells participate in Notch signaling by analyzing the expression of Hes5 mRNA via quantitative real−time PCR (qRT‒PCR); Hes5 is a gene that is activated by Notch signaling. Hes5 mRNA was detected in CD4^+^Notch2^+^ T cells but not in CD4^+^Notch2^−^ T cells. These findings indicate that Notch signaling is active in Notch2−expressing CD4^+^ T cells (Fig. 1D).

To investigate the inflammatory role of CD4^+^Notch2^+^ T cells in mice with EAE, we analyzed IL−17a and IFN−γ expression in CD4^+^Notch2^+^ T cells via flow cytometry (Fig. 1E–H). In addition, we used a cytokine bead array (CBA) to analyze the production of IL−17a and IFN−γ, both of which are proinflammatory cytokines closely associated with EAE symptoms (Fig. 1I, J). Both analyses revealed that Notch2−expressing CD4^+^ T cells expressed very low levels of IL−17a and IFN−γ. Because Notch2−expressing CD4^+^ T cells exhibit weak inflammatory properties, we hypothesized that they possibly have an immunoregulatory function during EAE progression. Next, we examined whether these cells are connected to the Treg cell subpopulation by measuring Foxp3 expression, as regulatory T cells expressing Foxp3 play a central role in immune regulation during the remission phase. For this analysis, we divided the EAE clinical phase into two stages−progression and remission−as shown in the schematic figure (Fig. 1K). Foxp3−expressing Treg cells were significantly expanded during the remission period. Interestingly, we found that CD4^+^Notch2^+^ T cells did not express Foxp3 during the progression phase, but we did observe low levels of Foxp3−expressing CD4^+^Notch2^+^ T cells during the remission phase (Fig. 1K–M). Accordingly, the Notch2−expressing population can be divided into Foxp3−negative (CD4^+^Notch2^+^Foxp3^−^) and Foxp3−low (CD4^+^Notch2^+^Foxp3^lo^) subsets, depending on the clinical stage. While the Foxp3−low population was identified among CD4^+^ cells infiltrating the spinal cords of EAE mice, it was not present in the inguinal lymph nodes or spleen of EAE mice during the remission stage (Supplementary Fig. 1A, B). In this analysis comparing Foxp3 levels in Notch2^+^ cells from the progression and remission stages of EAE, all Notch2^+^ cells were gated (Fig. 1L). However, in the characterization of CD4^+^Notch2^+^Foxp3^lo^ cells, Foxp3 levels were more strictly gated, as shown in Supplementary Fig. 1E.

To characterize CNS−infiltrated CD4^+^ T cells during EAE, we analyzed gene expression profiles at the single−cell level. For this analysis, to identify Notch2 protein surface expression, we used the Abseq surface labeling technique. Using single−cell RNA sequencing data, we performed sub−clustering of CD4^+^ T cells and classified them into eight distinct subclusters on the basis of shared gene expression markers (Fig. 1N, Supplementary Fig. 2A–C). Among these clusters, we identified a Notch2^+^Foxp3^lo^ population at the single−cell RNA sequencing level. To confirm that this cluster was of interest, we examined Notch2 protein expression and observed that the Notch2^+^Foxp3^lo^ cluster presented high Notch2 protein expression (Fig. 1O). To further characterize the heterogeneity of the CD4^+^ T−cell subclusters, we conducted trajectory analysis (Fig. 1P–R). By using UMAP−based annotation (Fig. 1N), we reconstructed the differentiation trajectories of CD4^+^ T−cell subclusters (Fig. 1P, Q). The analysis identified a naïve–like CD4^+^ T−cell cluster as the starting point of differentiation. Notably, the Treg cluster and Notch2^+^Foxp3^lo^ cluster represented distinct developmental trajectories, with Tregs branching earlier from an activated CD4^+^ T−cell state, whereas the Notch2^+^Foxp3^lo^ cluster emerged later along a separate trajectory. A dendrogram summarizing the differentiation pathways revealed six terminal states within the CD4^+^ T−cell subpopulations (Fig. 1R).

These findings highlight a unique differentiation pathway for the Notch2^+^Foxp3^lo^ cluster, which is distinct from conventional Treg development, suggesting a specialized role for Notch2 signaling in CD4^+^ T−cell heterogeneity and functional specialization.

### Foxp3−expressing CD4^+^Notch2^+^ T cells have immunomodulatory properties and exhibit a dysfunctional phenotype

**Fig. 2:**
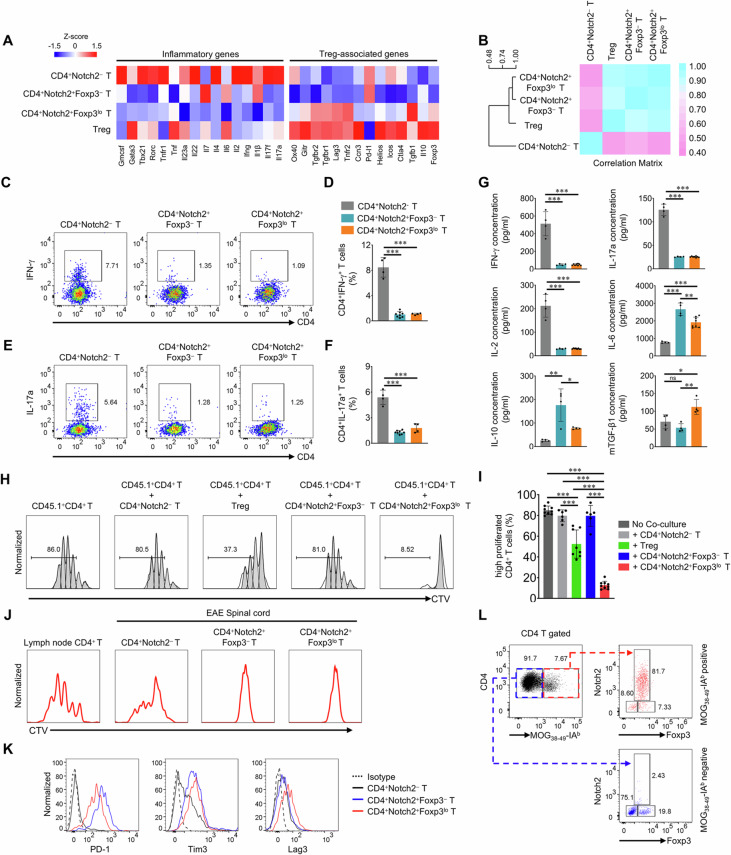
Foxp3−expressing CD4^+^Notch2^+^ T cells have immunomodulatory properties and exhibit a dysfunctional phenotype. Spinal cords were harvested 10−12 (P) or 21−25 (R) days after the induction of EAE. **A** RNA sequencing (RNA−seq) analysis of spinal cord−infiltrating CD4^+^ T cells was performed to evaluate the expression of representative RNAs encoding inflammatory factors and Treg cell−associated immunomodulatory factors. **B** Analysis of the correlation matrix via RNA sequencing (RNA−seq). **C** Representative flow cytometry analysis of IFN−γ^+^ cell populations within different populations of spinal cord-infiltrating CD4^+^ T cells. **D** Quantification of the IFN−γ^+^ cell populations via flow cytometry analysis (based on **C**). The graph shows the means ± SDs of the percentages. **E** Representative flow cytometry analysis of the IL−17a ^+^ cell populations within different populations of spinal cord-infiltrating CD4^+^ T cells. **F** Quantification of the results of flow cytometry analysis of the IL−17a^+^ cell populations (based on **E**). **G** Quantification of the cytokine bead array (CBA) results showing the production of several cytokines after TCR stimulation in different populations of spinal cord−infiltrating CD4^+^ T cells. The graph shows the means ± SDs of cytokine secretion. **H** Representative flow cytometry analysis of congenic CD4^+^ T−cell proliferation upon coculture with spinal cord−infiltrating CD4^+^ T cells. **I** Quantification of congenic CD4^+^ T−cell proliferation upon coculture with spinal cord−infiltrating CD4^+^ T cells (based on **H**). The graph shows the means ± SDs of high percentages of proliferating cells. **J** Representative flow cytometry analysis of proliferating CTV−labeled CD4^+^ T cells after stimulation for 72 h with anti−CD3 and anti−CD28 antibodies. **K** Representative flow cytometry analysis of protein expression (PD−1, Tim3, and Lag3) in CD4^+^Notch2^+ ^T cells from the spinal cords of EAE model mice. **L** Representative flow cytometry plots of MOG_38 − 49 _−specific tetramer−binding CD4^+ ^T cells from the spinal cords of EAE mice. The data are representative of two (**J**) or three (**C**−**Ι**, **Κ**, **L**) independent experiments (error bars, s.d. of > four (**D**, **F**, **G**, **I**) mice or samples). Statistical significance was calculated via one−way ANOVA followed by Tukey’s t−test for all the experiments; *P < 0.05, **P < 0.01, ***P < 0.001. ns, not significant

To assess the gene expression profile of each cell population (CD4^+^Notch2^−^ T cells, CD4^+^Notch2^+^Foxp3^−^ T cells, CD4^+^Notch2^+^Foxp3^lo^ T cells, and Treg cells), we conducted RNA−seq analysis. The results revealed that inflammation−related genes were expressed at lower levels in CD4^+^Notch2^+^ T cells than in CD4^+^Notch2^−^ T cells (Fig. 2A and Supplementary Fig. 3A–E). In addition, Treg−associated genes were highly expressed by Treg cells isolated from the spinal cord, whereas CD4^+^Notch2^+^Foxp3^lo^ T cells moderately expressed several Treg−associated genes. This similarity in gene expression was further assessed via correlation matrix analysis, which revealed that the gene expression profile of Notch2^+^ T cells was more similar to that of Treg cells than to that of Notch2^−^ T cells (Fig. 2B). Therefore, we investigated the expression of the Treg cell−related markers GITR, CLTA4, ICOS, and OX40 in CD4^+^Notch2^+^Foxp3^lo^ T cells to confirm whether they belong to the Treg cell population. However, the population expressed only OX40, the levels of which were only slightly increased in the Foxp3−expressing Notch2^+^ T−cell population (Supplementary Fig. 4A). These results indicate that CD4^+^Notch2^+^Foxp3^lo^ T cells do not represent a conventional Treg cell population.

Next, to determine whether Foxp3 expression is related to the properties of CD4^+^Notch2^+^ T cells, we compared CD4^+^Notch2^−^ T cells, CD4^+^Notch2^+^Foxp3^−^ T cells, and Foxp3−expressing CD4^+^Notch2^+^ T cells in terms of the production of IFN−γ and IL−17a. The flow cytometry results revealed that Notch2−expressing CD4^+^ T cells exhibited very low production of IFN−γ and IL−17a, regardless of Foxp3 expression (Fig. 2C–F), a finding that was confirmed by the CBA results (Fig. 2G). We also analyzed the production of IL−2 and IL−6 and found that IL−2 was rarely produced by Notch2−expressing CD4^+^ T cells. Additionally, IL−6 was produced at higher levels by CD4^+^Notch2^+^Foxp3^−^ T cells than by CD4^+^Notch2^−^ T cells, whereas Foxp3−expressing CD4^+^Notch2^+^ T cells produced less IL−6 than did CD4^+^Notch2^+^Foxp3^−^ T cells (Fig. 2G). Thus, Foxp3 expression significantly reduces IL−6 levels in CD4^+^Notch2^+^ T cells.

We also analyzed the production of the immunomodulatory cytokines IL−10 and TGF−β. Notch2−expressing CD4^+^ T cells produced more IL−10 than did CD4^+^Notch2^−^ T cells upon TCR stimulation. Moreover, only CD4^+^Notch2^+^Foxp3^lo^ T cells presented a significant increase in TGF−β production (Fig. 2G). To assess the inhibitory function of activated−T cell proliferation, congenic CD45.1^+^CD4^+^CD25^−^ T cells were cocultured with each type of spinal cord−infiltrating CD45.2^+^CD4^+^ T−cell (i.e., CD4^+^Notch2^−^ T cells, CD4^+^Notch2^+^Foxp3^−^ T cells, CD4^+^Notch2^+^Foxp3^lo^ T cells or Tregs isolated from the spinal cords of EAE mice) under TCR/CD28 stimulation via anti−CD3/CD28−coated beads. In this coculture experiment, Foxp3−expressing CD4^+^Notch2^+^ T cells more strongly inhibited activated CD4^+^ T−cell proliferation than Tregs did (Fig. 2H, I). This effect was not affected by TNFα blockade (Supplementary Fig. 5A).

CD4^+^Notch2^+^ T cells secreted fewer inflammatory cytokines than did CD4^+^Notch2^−^ T cells. We speculated that this was due to an activation problem; therefore, we analyzed the expression of activation markers by CD4^+^ T cells that infiltrate the spinal cords of EAE mice. The expression of CD69, CD44, and CD25 on Notch2−expressing CD4^+^ T cells (CD4^+^Notch2^+^Foxp3^−^ T cells and CD4^+^Notch2^+^Foxp3^lo^ T cells) was induced upon TCR/CD28 stimulation (Supplementary Fig. 6A, B). However, the proliferation of Notch2−expressing T cells was much lower than that of Notch2−negative T cells, which suggests that the population exhibited a dysfunctional phenotype (Fig. 2J). CD4^+^Notch2^−^ T cells from both the progression and remission stages presented similar levels of CD44 and CD69, indicating that they maintain consistent effector functions throughout the EAE stages (Supplementary Fig. 6A). Because the population exhibited a nonproliferative phenotype possibly similar to an exhausted phenotype, we examined the expression of PD−1, Tim3, and Lag3 (representative markers of nonproliferating, exhausted CD8^+ ^T cells) [30]. The results revealed that CD4^+^Notch2^+^ T cells expressed high levels of PD−1, Tim3, and Lag3 (Fig. 2K). We examined the expression of the Tox and Nr4A genes in our RNA−seq data. In the Tox family, the expression levels of Tox1, Tox2, and Tox3 were lower than those of Tox4 and the Nr4A family in infiltrating CD4^+^ T cells. However, the expression levels of these genes did not differ between CD4^+^Notch2^+^ T cells and CD4^+^Notch2^−^ T cells (Supplementary Fig. 6C), suggesting that these dysfunctional CD4^+^Notch2^+^ T cells may not be part of the conventional exhausted T−cell population. Additionally, nearly all CD4^+^Notch2^+^Foxp3^lo^ T cells were MOG−specific (Fig. 2L), suggesting that the antigen−specific cells in the inflamed CNS exhibited a dysfunctional phenotype and that Notch2 was expressed in this population.

### CD4^+^Notch2^+^Foxp3^lo^ T cells attenuate EAE symptoms

**Fig. 3:**
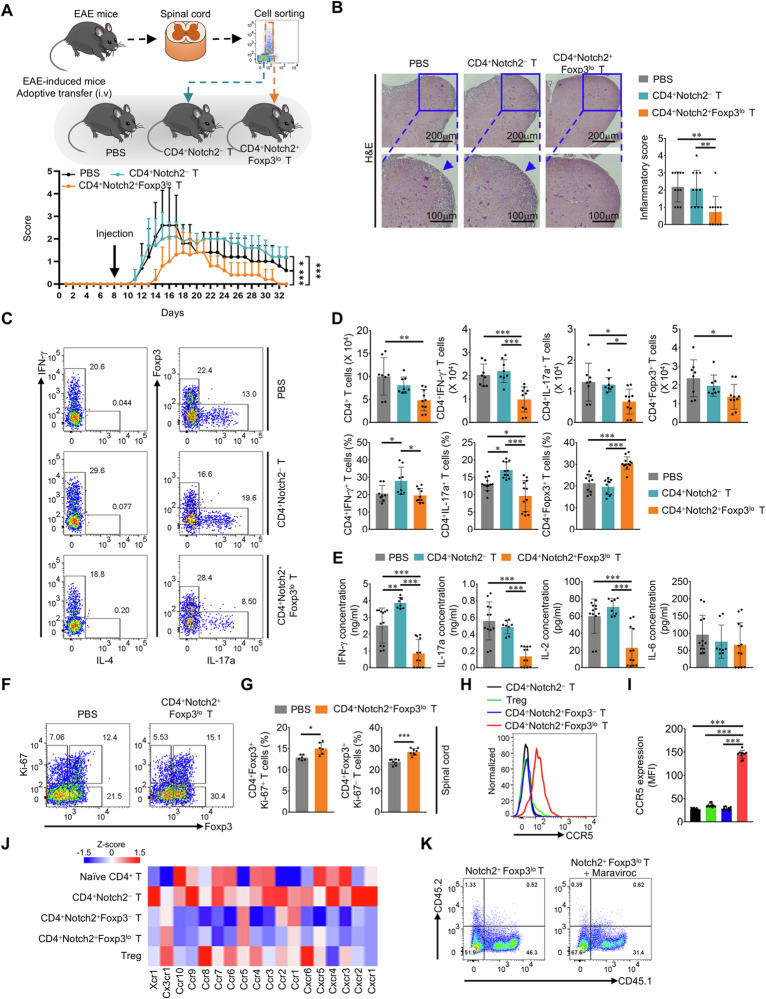
CD4^+^Notch2^+^Foxp3^lo^ T cells attenuate EAE symptoms. EAE was induced in C57BL/6 J and *Foxp3*^eGFP^ mice, and CD4^+^Notch2^−^T cells and CD4^+^Notch2^+^Foxp3^lo^ T cells were isolated from the spinal cords 10−12 (P) or 21−25 (R) days after the induction of EAE. Different types of isolated CD4^+^ T cells were injected intravenously into EAE model C57BL/6 J mice. **A** Experimental plan (upper) and clinical scores (lower) of EAE mice that underwent adoptive transfer of isolated spinal cord − infiltrating CD4^+^ T cells. The graph shows the means ± SDs of the EAE clinical scores. **B** H&E staining and inflammatory scores of lumbar spinal cord tissue from EAE model mice subjected to adoptive transfer. **C** Representative flow cytometry analysis of IFN−γ^+^ , IL−17a^+^ , and Foxp3^+^CD4^+^ T cells in the spinal cords of EAE model mice subjected to adoptive transfer. **D** Quantification of spinal cord−infiltrating IFN−γ^+^ , IL−17a^+^ , and Foxp3^+^CD4^+^ T cells in EAE model mice subjected to adoptive transfer (based on **C**). The graph shows the means ± SDs of the cell numbers and percentages. **E** Quantification of inflammatory cytokine production in EAE mice subjected to adoptive transfer. The graph shows the means ± SDs of cytokine secretion. **F** Representative flow cytometry analysis of proliferating and Foxp3−expressing CD4^+^ T cells in the spinal cords of C57BL/6 J mice subjected to adoptive transfer. **G** Quantification of proliferating and Foxp3−expressing CD4^+^ T cells (based on **F**). The graph shows the means ± SDs of the percentage of cells. **H** Representative flow cytometry analysis of CCR5 expression on the cell surface. **I** Quantification of CCR5 expression (based on **H**). The graph shows the mean ± SD of the CCR5 MFI value. **J** Heatmap showing various chemokine receptor genes from the bulk RNA−seq dataset. **K** Representative flow cytometry analysis of CD45.1^+^ and CD45.2^+^CD4^+^ T cells among total spinal cord−infiltrating cells following treatment with maraviroc, a CCR5 inhibitor. CD45.2^+^CD4^+^Notch2^+^Foxp3^lo^ T cells were adoptively transferred into CD45.1^+^ recipient mice. Maraviroc was dissolved in polyethylene glycol 400 (PEG400) and administered intraperitoneally one day prior to adoptive transfer. The data are representative of two (**E**–**I**) or three (**A**−**D**, **K**) independent experiments (error bars, s.d. of six (**G**) or > eight (**A**, **B**, **D**, **E**, **I**) mice or samples). Statistical significance was calculated via two−way ANOVA followed by Tukey’s multiple comparison in (**A**); one−way ANOVA followed by Tukey’s multiple comparison in (**B**, **D**, **E**, **I**); and two−way ANOVA followed by an unpaired t−test in (**G**); *P < 0.05, **P < 0.01, ***P < 0.001

To verify the immune modulatory functions of CD4^+^Notch2^+^Foxp3^lo^ T cells in vivo, we adoptively transferred these cells from EAE−induced *Foxp3*^eGFP^ mice (CD45.2) into EAE mice (CD45.1). Compared with mice injected with phosphate−buffered saline (PBS) or CD4^+^Notch2^−^ T cells, mice receiving CD4^+^Notch2^+^ Foxp3^lo^ T cells had less severe disease, and histological analysis revealed reduced infiltration of cells into the spinal cord as well as decreased damage (Fig. 3A, B). Next, we examined IFN−γ−, IL−17a−, and Foxp3−expressing CD4^+^ T cells in the inguinal lymph nodes, spleens, and spinal cords. In the periphery, there was no marked difference between the CD4^+^Notch2^+^Foxp3^lo^ T−cell group and the PBS group with respect to cell populations (Supplementary Fig. 7A). In the spleen, the transfer of CD4^+^Notch2^−^ T cells led to a significant increase in the total number of CD4^+^ T cells (Supplementary Fig. 7A). Additionally, in the inguinal lymph nodes, the percentage of proliferating Ki−67−positive Treg cells was increased in the mice that received CD4^+^Notch2^+^Foxp3^lo^ T cells (Supplementary Fig. 7B). Consistent with the histological analysis results, the transfer of CD4^+^Notch2^+^Foxp3^lo^ T cells significantly reduced the number of CD4^+^ T cells infiltrating the spinal cord, as did the number of IFN−γ− and IL−17a−producing cells (Fig. 3C, D). The transferred CD45.2 congenic CD4^+^Notch2^+^Foxp3^lo^ T cells were found primarily in the CNS region and not in the periphery (Supplementary Fig. 8A–C). Interestingly, the percentage of Treg cells among infiltrated CD4^+^ T cells in the CNS region was greater following the transfer of CD4^+^Notch2^+^Foxp3^lo^ T cells (Fig. 3D). Additionally, the production of IFN−γ, IL−17a, and IL−2 by spinal cord−infiltrating cells was markedly decreased by this transfer (Fig. 3E). As the Treg population was increased by the transfer of CD4^+^Notch2^+^Foxp3^lo^ T cells, we examined proliferating Ki−67−positive Treg cells. The results revealed that the percentage of proliferating Ki−67−positive Treg cells in CNS regions was greater in CD4^+^Notch2^+^Foxp3^lo^ T cell−transferred EAE mice (Fig. 3F, G). In our experimental system, the transfer of CD4^+^Notch2^+^Foxp3^lo^ T cells did not significantly regulate the inflammatory population in the periphery, but the population in the CNS was regulated by transfer. This result may be attributed to the high level of CCR5 expression on CD4^+^Notch2^+^Foxp3^lo^ T cells (Fig. 3H–J), allowing them to migrate to inflamed regions [31–33]. To clarify the crucial role of CCR5, we performed adoptive transfer of CD45.2^+^CD4^+^Notch2^+^Foxp3^lo^ T cells into CD45.1 mice treated with the CCR5 inhibitor maraviroc. As a result, infiltration of the transferred cells into the CNS was blocked by the inhibitor treatment (Fig. 3K).

### Deletion of Notch2 expression in Foxp3−expressing cells prolongs EAE symptoms

**Fig. 4:**
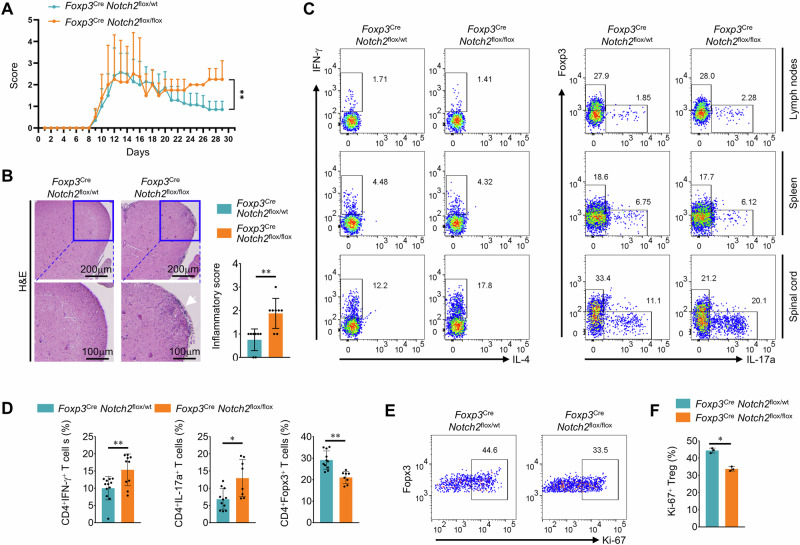
Deletion of Notch2 expression in Foxp3−expressing cells prolongs EAE symptoms. EAE was induced in *Foxp3*^YFP − Cre^*:Notch2*^flox/wt^ and *Foxp3*^YFP − Cre^*:Notch2*^flox/flox^ mice. **A** Clinical EAE scores of *Foxp3*^YFP − Cre^*:Notch2*^flox/wt^ and *Foxp3*^YFP − Cre^*:Notch2*^flox/flox mice^. The graph shows the means ± SDs of the EAE clinical scores. **B** H&E staining and inflammatory scores of spinal cords from *Foxp3*^YFP − Cre^*:Notch2*^flox/wt^ and *Foxp3*^YFP − Cre^*:Notch2*^flox/flox^ EAE model mice. **C** Representative flow cytometry analysis of IFN−γ^+^ , IL−17a^+^ , and Foxp3^+^ CD4^+^ T cells in the lymph nodes, spleens, and spinal cords of EAE mice. **D** Quantification of IFN−γ^+^ , IL−17a^+^ , and Foxp3^+^CD4^+^ T cells in the spinal cord (based on **C**). The graph shows the means ± SDs of the percentage of cells. **E** Representative flow cytometry analysis of the proliferation of spinal cord−infiltrating Treg cells on the basis of the expression of Ki−67. **F** Quantification of Ki−67−expressing Tregs (based on **E**). The graph shows the means ± SDs of the percentage of Ki−67^+^ Treg cells. The data are representative of two (**B**, **E**, **F**) or three (**A**, **C**, **D**) independent experiments (error bars, s.d. of three (**F**), eight (**B**, **D**), or nine (**A**) mice). Statistical significance was calculated via two−way ANOVA followed by an unpaired t−test for all the experiments; *P < 0.05, **P < 0.01

To examine whether CD4^+^Notch2^+^Foxp3^lo^ T cells play a direct role in alleviating EAE symptoms, we compared wild−type (WT; *Notch2*^flox/wt^: *Foxp3*^YFP − Cre^) and conditional knockout (cKO; *Notch2*^flox/flox^: *Foxp3*^YFP − Cre^) mice with EAE mice. The disease incidence in EAE−induced cKO mice was not different from that in WT mice (data not shown). However, the cKO mice presented prolonged and severe EAE symptoms (Fig. 4A). Histological analysis of spinal cord tissues during the remission phase revealed lower levels of infiltration in WT mice than in cKO mice (Fig. 4B). Consistent with these findings, flow cytometry analysis revealed that the numbers of CD4^+^IFN−γ^+^ T cells and CD4^+^IL−17a^+^ T cells in cKO mice were greater than those in WT mice, although the total number of CD4^+^ T cells was similar, and there were fewer Treg cells in the spinal cords of cKO mice (Fig. 4C, D). In addition, Foxp3−expressing Treg cells in the spinal cords of cKO mice exhibited reduced proliferation, as the percentage of proliferating Ki−67−positive Treg cells was reduced (Fig. 4E, F). Overall, CD4^+^Notch2^+^Foxp3^lo^ T cells support Treg cell expansion, thereby promoting recovery from autoimmune diseases.

### Notch2 expression is crucial for CD4^+^Notch2^+^Foxp3^lo^ T cell−mediated expansion of Treg cells

**Fig. 5:**
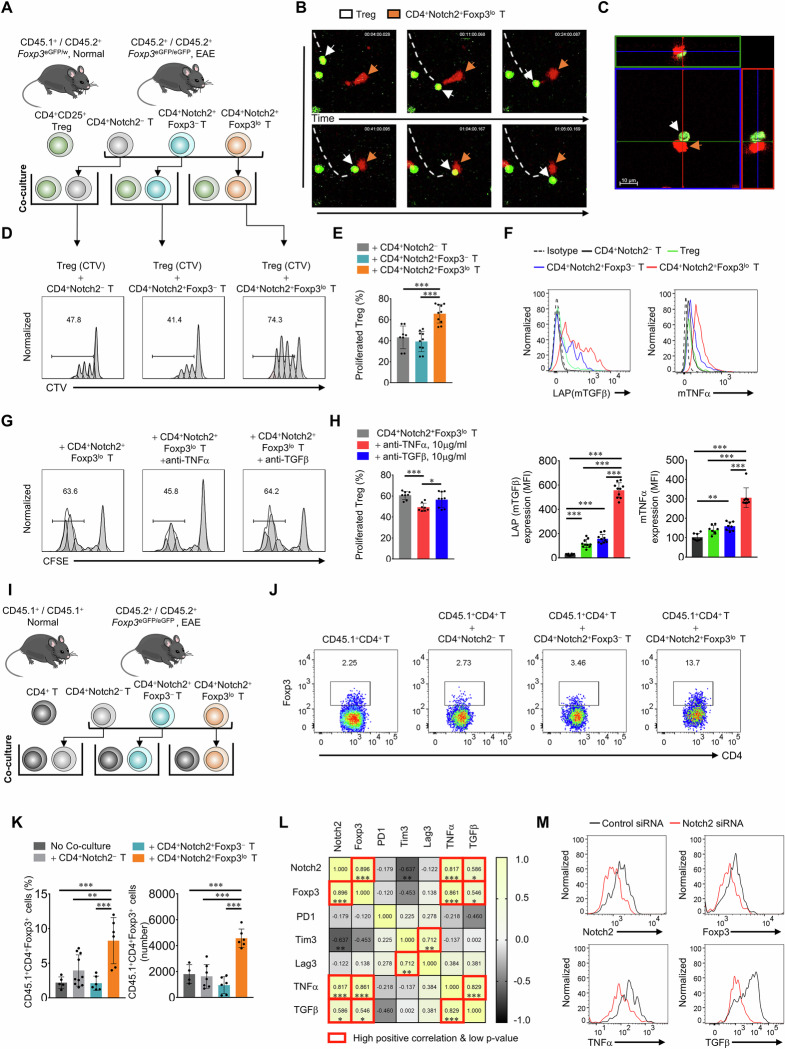
Notch2 is crucial for CD4^+^Notch2^+^Foxp3^lo^ T cell−mediated expansion of Treg cells. EAE was induced in C57BL/6 J and *Foxp3*^eGFP^ mice. Spinal cords were harvested at 10−12 (P) or 21−25 (R) days postinduction of EAE. Spinal cord−infiltrating CD4^+^ T cells isolated from EAE model mice were cocultured with congenic naïve CD4^+^ T cells or Treg cells. Tregs were isolated from the spinal cord via FACS−sorting. **A** Schematic showing the in vitro coculture experiment using Treg cells. **B** Confocal live imaging of Treg and CD4^+^Notch2^+^Foxp3^lo^ T cell cocultures (Treg:CD4^+^Notch2^+^Foxp3^lo^ T cells at a 3:1 ratio). **C** 3D imaging of Treg and CD4^+^Notch2^+^Foxp3^lo^ T cell cocultures. **D** Representative flow cytometry analysis of in vitro Treg cell proliferation upon coculture with spinal cord−infiltrating CD4^+^ T cells for 72 h. **E** Quantification of Treg proliferation (based on **D**). The graph shows the means ± SDs of the percentages of proliferated Tregs. **F** Representative flow cytometry analysis of membrane−bound TGF−β (LAP) and membrane−bound TNFα expression in the spinal cord−infiltrating CD4^+^ T cells. The graph shows the means ± SDs of the cytokine MFI values. **G** Representative flow cytometry histogram showing the proliferation of peripheral Treg cells in the presence/absence of TNFα and TGF−β neutralizing antibodies (10 μg/ml). **H** Quantification of Treg proliferation (based on **G**). The graph shows the means ± SDs of the percentages of proliferated Tregs. **I** Schematic showing the in vitro coculture experiment using naïve CD4^+^ T cells. **J** Differentiation of congenic CD4^+^ T cells into iTreg cells upon coculture with spinal cord−infiltrating CD4^+^ T cells without TGF−β for 72 h. **K** Quantification of the percentage and number of iTregs differentiated (based on **J**). The graph shows the means ± SDs of the percentage of Foxp3^+^−expressing cells. **L** Correlation analysis plot of flow cytometry MFI values for CD4^+^Notch2^+^Foxp3^lo^ T cells after Notch2 siRNA treatment. **M** Representative flow cytometry analysis of the effects of Notch2 siRNA or control siRNA treatment on CD4^+^Notch2^+^Foxp3^lo^ T cells. The data are representative of two (**L**, **M**) or three (**B**−**K**) independent experiments (error bars, s.d. of > five (**K**), six (**L**), or > eight (**E**, **F**, **H**) mice or samples). Statistical significance was calculated via one−way ANOVA followed by Tukey’s multiple comparison in (**E**, **F**, **H**, **K**) and the correlation matrix in (**L**); *P < 0.05, **P < 0.01, ***P < 0.001

During the remission stage of EAE, the number of Treg cells markedly increased, as did the number of CD4^+^Notch2^+^Foxp3^lo^ T cells. Additionally, adoptive transfer of CD4^+^Notch2^+^Foxp3^lo^ T cells increased the number of Treg cells in the inflamed CNS. Therefore, we examined whether CD4^+^Notch2^+^Foxp3^lo^ T cells play a role by directly affecting Treg cells through a coculture experiment (Fig. 5A). In the coculture experiment, we found that Treg cells directly interacted with CD4^+^Notch2^+^Foxp3^lo^ T cells when we examined the interaction via a live imaging system (Fig. 5B, C and Supplementary Video. 1). In addition, CD4^+^Notch2^+^Foxp3^lo^ T cells (CD45.2) increased the proliferation of isolated CD4^+^Foxp3^eGFP^CD25^+^ Treg cells (CD45.1/CD45.2) to a greater extent than did CD4^+^Notch2^−^ T cells (CD45.2) and CD4^+^Notch2^+^Foxp3^−^ T cells (CD45.2) (Fig. 5D, E). Notably, the expression of both forms of TNFα (soluble and membrane−bound), as well as that of TGF−β, by CD4^+^Notch2^+^Foxp3^lo^ T cells was high (Figs. 2G, 5F and Supplementary Fig. 9A); both of these cytokines play a role in expanding the Treg cell population and Treg cell differentiation. Interestingly, neither Foxp3−expressing Treg cells nor CD4^+^Notch2^+^Foxp3^−^ T cells function as signaling cells in TGF−β− and TNFα−mediated juxtacrine interactions (Fig. 5F). Only cells co−expressing both Notch2 and Foxp3 exhibit high levels of membrane−bound TNFα and TGF−β. We hypothesize that one of these two membrane−bound cytokines predominantly drives juxtacrine signaling to promote Treg cell proliferation. As is well known, membrane−bound TNFα specifically activates TNF receptor 2 (TNFR2) on Treg cells, resulting in Treg cell proliferation [34]. Our results also revealed that CNS−infiltrated Treg cells expressed both TNFR1 and TNFR2 (Supplementary Fig. 10A). Consistent with these findings, neutralization assays revealed that coculture of CD4^+^Notch2^+^Foxp3^lo^ T cells with an anti−TNFα− blocking antibody led to a marked reduction in the expansion of the CD4^+^Notch2^+^Foxp3^lo^ T cell−mediated Treg cell population (Fig. 5G, H). We also examined whether CD4^+^Notch2^+^ T cells play a role in Treg differentiation. Congenic CD45.1^+^CD4^+^CD25^−^ T cells were cocultured with each type of spinal cord−infiltrating CD45.2^+^CD4^+^ T cells (i.e., CD4^+^Notch2^−^ T cells, CD4^+^Notch2^+^Foxp3^−^ T cells or CD4^+^Notch2^+^Foxp3^lo^ T cells isolated from the spinal cords of EAE−induced mice) under TCR/CD28 stimulation conditions (Fig. 5I). CD4^+^Notch2^+^Foxp3^lo^ T cells (CD45.2) induced marked differentiation of Foxp3^+^−induced Treg cells (CD45.1) without TGF−β (Fig. 5J, K). In addition, we found that the ability of CD4^+^Notch2^+^Foxp3^lo^ T cells to stimulate Treg cells was dependent on Notch2 expression. When Notch2 expression was regulated by a small interfering (si)RNA against Notch2 mRNA, the reduction in Notch2 expression varied across the samples. In this study, we analyzed the correlation of Notch2 expression with that of PD−1, Tim3, Lag3, Foxp3, TNFα, and TGF−β., while the expression of Foxp3, TNFα, and TGF−β was significantly positively correlated with that of Notch2, whereas the expression of PD−1, Tim3, and Lag3 was not (Fig. 5L, M). Taken together, these findings underscore the influence of Notch2 expression on Foxp3 regulation and reveal that the co−expression of Notch2 and Foxp3 evokes TNFα and TGF−β membrane expression.

### Notch2 expression is regulated by the E3 ubiquitin ligase Nedd4

**Fig. 6:**
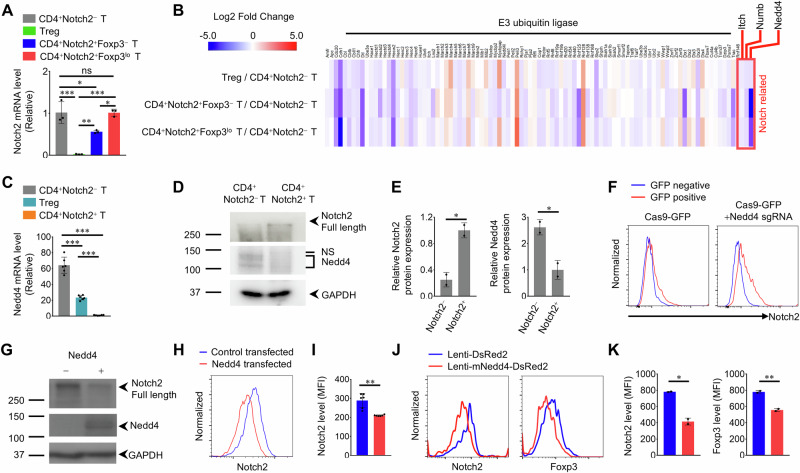
Notch2 expression is regulated by the E3 ubiquitin ligase Nedd4. EAE was induced in C57BL/6 J and *Foxp3*^eGFP^ mice. Spinal cords were harvested 10−12 (P) or 21−25 (R) days postinduction. **A** qRT−PCR analysis of Notch2 mRNA expression in different types of spinal cord−infiltrating CD4^+^ T cells. The graph shows the means ± SDs of the relative Notch2 mRNA levels. **B** Expression analysis of genes encoding E3 ubiquitin ligases in different types of spinal cord−infiltrating CD4^+^ T cells from the bulk RNA−seq dataset, displayed as a heatmap. **C** qRT−PCR analysis of Nedd4 mRNA expression in different types of spinal cord−infiltrating CD4^+^ T cells. The graph shows the means ± SDs of the relative Notch2 mRNA levels. **D** Western blot analysis of Notch2 and Nedd4 protein expression in CD4^+^Notch2^−^ T cells and CD4^+^Notch2^+^ T cells. **E** Quantification of protein expression (based on **D**). The graph shows the means ± SDs of relative protein expression. **F** Representative flow cytometry analysis of surface Notch2 expression in Nedd4 sgRNA and Cas9 RNP electroporated CD4^+^Notch2^−^ T cells. **G** Western blot analysis of Notch2 protein expression in HEK293T cells overexpressing Nedd4. **H** Representative flow cytometry analysis of surface Notch2 expression in HEK293T cells overexpressing Nedd4. **I** Quantification of the Notch2 MFI (based on **H**). The graph shows the mean ± SD of the Notch2 MFI. **J** Representative flow cytometry analysis of Notch2 and Foxp3 expression in Nedd4−transduced CD4^+^Notch2^+^Foxp3^lo^ T cells. **K** Quantification of the MFI (based on **J**). The data are representative of two (**C**, **F**, **G**) or three (**A**, **D**, **E**, **H**−**K**) independent experiments (error bars, s.d. of two (**E**, **K**), three (**A**), or six (**C**, **I**) samples or mice). Statistical significance is calculated via one−way ANOVA followed by Tukey’s multiple comparison in (**A**, **C**), unpaired two−tailed Student’s t test in (**E**), and two−way ANOVA followed by unpaired t−test in (**I**, **K**); *P < 0.05, **P < 0.01, ***P< 0.001. ns, not significant

Notch2^+ ^T cells presented significantly greater expression of the Notch2 protein than did Notch2^−^ T cells (Fig. 1A). However, the expression of Notch2 mRNA did not differ between CD4^+^Notch2^−^ T cells and CD4^+^Notch2^+^Foxp3^lo^ T cells (Fig. 6A). Thus, we hypothesized that the expression of the Notch2 receptor is regulated posttranscriptionally. Previous studies have shown that the expression of several E3 ubiquitin ligases is strongly correlated with Notch protein regulation [35–37]. Indeed, analysis of Notch−related E3 ubiquitin ligase RNA−seq data revealed that Notch2^+^ cells expressed much lower levels of Nedd4 than did Notch2^−^ cells (Fig. 6B). Nedd4 is an E3 ubiquitin ligase that ubiquitinates membrane proteins such as ion channels and receptors [38] to mediate membrane protein endocytosis [39] and inflammation [40].

A previous study revealed that Notch protein degradation is mediated by Nedd4 [37]. To examine the expression of Nedd4 in specific cells, such as Treg cells, Notch2^−^ cells, and Notch2^+ ^cells, we measured the expression of Nedd4 mRNA via qRT−PCR. As expected, the expression of Nedd4 mRNA was much greater in Notch2^−^ cells than in Notch2^+^ cells or Treg cells (Fig. 6C). We also performed Western blotting to confirm the protein expression of Nedd4. Consistent with the mRNA results, Nedd4 protein expression was greater in Notch2^−^ cells than in Notch2^+^ cells (Fig. 6D, E). Moreover, flow cytometry analysis revealed that Cas9 and sgRNA mediated deletion of *Nedd4* upregulated Notch2 expression in CD4^+^Notch2^−^ T cells (Fig. 6F and Supplementary Fig. 11A). Additionally, compared with control cells, HEK293T cells transfected with Nedd4 in vitro expressed less Notch2 protein (Fig. 6G–I). Furthermore, CD4^+^Notch2^+^Foxp3^lo^ T cells transduced with Nedd4 via a recombinant lentivirus and isolated from the spinal cords of EAE mice presented lower expression of Notch2 than did CD4^+^Notch2^+^Foxp3^lo^ T cells transfected with a control lentivirus (Fig. 6J, K). Consistent with the siRNA−mediated decrease in Notch2 expression (Fig. 5L, M), Foxp3 expression was also reduced by Nedd4 expression along with that of Notch2 (Fig. 6J, K).

To confirm that these results were not affected by other proteasome subunits, we examined the expression of these subunits via RNA−seq. The CD4^+^Notch2^+^ and CD4^+^Notch2^−^ T cell populations did not show any marked differences with respect to the expression of mRNAs encoding proteasome subunits (Supplementary Fig. 12A). Thus, we concluded that Nedd4 is the key regulator of Notch2 expression in CNS−infiltrating CD4^+^ T cells.

### CD4^+^Notch2^+^Foxp3^lo^ T cells are present in both DSS−induced colitis model mice and humans with UC

**Fig. 7:**
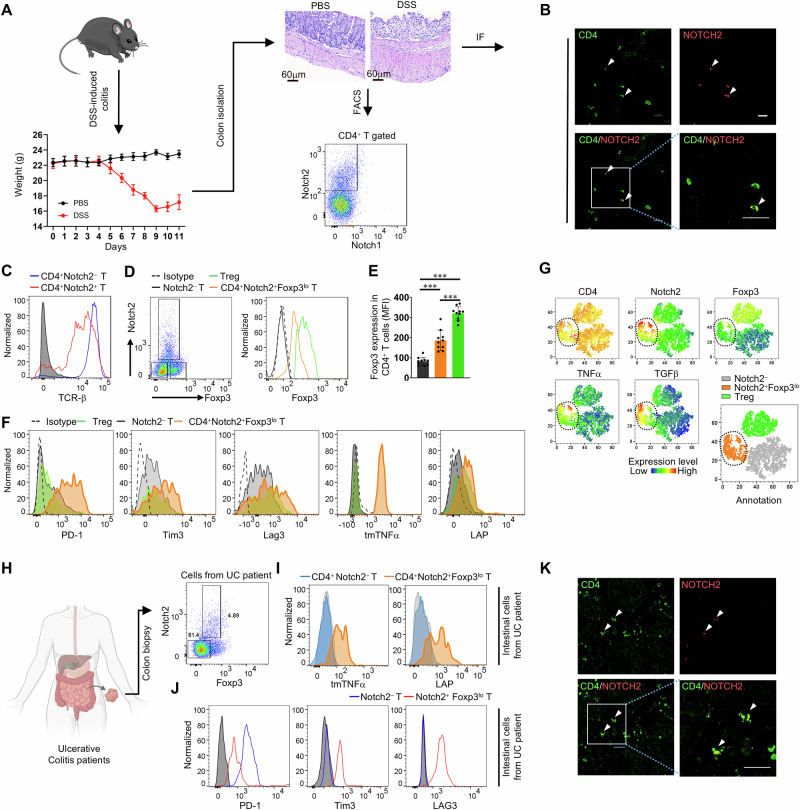
CD4^+^Notch2^+^Foxp3^lo^ T cells are present in both DSS−induced colitis model mice and humans with UC. DSS−induced colitis was induced in C57BL/6 J mice. **A** Weights of the mice with DSS−induced colitis (left bottom panel). H&E staining of colon tissues from DSS−induced colitis model mice (right upper panel). Representative flow cytometry analysis of Notch1 and Notch2 expression in CD4^+^ T cells infiltrating the colons of mice with colitis (right bottom panel). **B** Representative immunofluorescence analysis of CD4 (green) and Notch2 (red) expression in the colons of DSS−induced mice. Scale bar: 20 μm. **C** Representative flow cytometry analysis of TCR−β expression by CD4^+^ T cells infiltrating the colons of mice with colitis. **D** Representative flow cytometry analysis of Notch2 and Foxp3 expression in colon−infiltrating CD4^+^ T cells. **E** Quantification of the MFI (based on **D**). The graph shows the mean ± SD of the Foxp3 MFI. **F** Representative flow cytometry analysis of protein expression (PD−1, Tim3, and Lag3) in CD4^+^Notch2^+^Foxp3^lo^ T cells in the colons of colitis model mice. Flow cytometry analysis of membrane−bound TGF−β (LAP) and membrane−bound TNFα expression by CD4^+^Notch2^+^Foxp3^lo^ T cells from the colons of mice with DSS−induced colitis was performed. **G** CD4^+^ T cells are projected into the t−SNE space, with the two t−SNE components shown as the axes of the plot. **H** Representative flow cytometry analysis of Notch2 and Foxp3 expression in colon−infiltrating CD4^+^ T cells in biopsy samples from patients with IBD. A schematic illustration was generated via BioRender. Bae, S. (2025) https://BioRender.com/koj9yz4. **I** Representative flow cytometry analysis of membrane−bound TGF−β (LAP) and membrane−bound TNFα expression by CD4^+^Notch2^+^Foxp3^lo^ T cells from the colons of patients with IBD. **J** Representative flow cytometry analysis of protein expression (PD−1, Tim3, and Lag3) in CD4^+^Notch2^+^Foxp3^lo^ T cells from the colons of patients with IBD. **K** Representative immunofluorescence analysis of CD4 (green) and Notch2 (red) expression in the colons of UC patients. Scale bar: 20 μm. The data are representative of two (**G**), three (**A**−**F**) or seven (**H**−**K**) independent experiments (error bars, s.d. of ten (**E**) samples). Statistical significance was calculated via one−way ANOVA followed by Tukey’s multiple comparison test in (**E**) experiments; ***P < 0.001

Next, we investigated whether this cell population was also associated with other inflammatory conditions. To test this hypothesis, we used a DSS−induced colitis mouse model, which has clinical characteristics that resemble those of human UC [41]. We found that intestinal inflammation was successfully induced under our experimental conditions (Fig. 7A), as we observed both weight loss and remission (Fig. 7A). As observed in the EAE model, we also identified CD4^+^Notch2^+^ T cells in the lamina propria region of the colitis model mice (Fig. 7A, B); this population also expressed the TCR (Fig. 7C). However, none of the CD4^+^ T cell populations in this region expressed Notch1. Like CD4^+^Notch2^+^Foxp3^lo^ T cells in EAE model mice, Notch2−expressing CD4^+^ Τ cells also expressed low levels of Foxp3 (Fig. 7D, E). Furthermore, these CD4^+^Notch2^+^Foxp3^lo^ T cells expressed PD−1, along with low levels of Tim3 and Lag3 (Fig. 7F). Most importantly, the CD4^+^Notch2^+^Foxp3^lo^ T cells in the colitis model mice also expressed membrane−bound TNFα and TGF−β (LAP) (Fig. 7F). In addition, CD4^+^Notch2^+^Foxp3^lo^ T cells could be discriminated by a reduction in t−SNE−based dimensionality (Fig. 7G). Taken together, these findings demonstrate that CD4^+^Notch2^+^Foxp3^lo^ T cells contribute to inflammation associated with various autoinflammatory diseases. We also examined the population of cells in the lamina propria by analyzing freshly prepared intestinal biopsy samples from UC patients in clinical remission or those with mild or moderate inflammation (Supplementary Fig. 13A). Strikingly, this cell population in the patient lamina propria (Fig. 7H–Κ and Supplementary Fig. 13A–C) was phenotypically similar to the CD4^+^Notch2^+^Foxp3^lo^ T cell population observed in the EAE and DSS mouse models. In humans, CD4^+^Notch2^+^Foxp3^lo^ T cells express functional receptors (i.e., membrane−bound TNFα and TGF−β), as well as molecules consistent with the dysfunctional phenotype − similar to exhaustion, characterized by a nonproliferative state and expression of PD−1, Tim3, and Lag3−, as observed in mouse models (Fig. 7I).

## Discussion

Recent studies have attempted to optimize low−dose IL−2 therapy to activate Treg cells specifically [42] or expand Treg cells (from patients)−based on cell therapy to patients with autoimmune disease [43]; however, challenges remain, particularly with respect to difficulties associated with in vitro and ex vivo expansion and purification [44]. The results presented in the present study may facilitate progress in the development of next−generation Treg cell−based therapies. A recent study revealed that Foxp3−specific Notch2 KO mice harbor low numbers of regulatory T cells [45, 46]. Research on in vitro Treg differentiation using human CD4^+^ T cells revealed that Notch2 overexpression increased Treg numbers [47]. It is difficult to directly compare these previous studies with our current work because of differences in experimental systems. Therefore, our results offer tentative support for the theory that Notch2 expression influences Treg functionality. Previous reports suggest that Jag1 is associated with the acquisition of immune regulatory functions in CD4^+^ T cells [48–50] and is also linked to immune evasion within the tumor microenvironment [51]. Although Notch1 and Notch2 are the main receptors for Jag1, the ligand has a relatively high affinity for Notch2 [52]. Our preliminary results revealed that, among the Notch ligands, the expression of Jag1 increased mainly in the CNSs of EAE mice. In addition, among infiltrating CD4^+^ T cells, Notch2−expressing CD4^+^ T cells are present in the spinal cord and express Foxp3 during the remission phase of EAE. Thus, the Jag1‒Notch2 axis in CD4^+^ T cells may be associated with immunomodulation. Consistent with this idea, dysfunctional CD4^+^Notch2^+^Foxp3^lo^ T cells are nonproliferative; express PD−1, Tim3, and Lag3; and exhibit decreased inflammatory functions, such as reduced expression of IFN−γ and IL−17a. Eventually, this population expressed low levels of Foxp3 and exhibited a more immunoregulatory phenotype than did the Foxp3−negative population. Rather than expressing these inflammatory cytokines, the population expressed higher levels of TGF−β and IL−10, both of which are involved in remission from EAE symptoms [53], than the CD4^+^Notch2^−^ T cell population. In addition, CD4^+^Notch2^+^Foxp3^lo^ T cells inhibited the proliferation of activated CD4^+^ T cells. However, TNFα did not affect suppressive activity. CD4^+^Notch2^+^Foxp3^−^ T cells displayed reduced inflammatory features but still expressed the inflammatory cytokine IL−6. However, during the remission phase of EAE, CD4^+^Notch2^+^ T cells expressed low levels of Foxp3, whose expression was dependent on Notch2; nevertheless, they differed from conventional Treg cells with respect to the expression of Treg cell markers such as GITR, CTLA−4, ICOS, and OX40, suggesting that they do not belong to the general Treg cell population. Even though this population does not belong to the general Treg population, adoptive transfer of CD4^+^Notch2^+^Foxp3^lo^ T cells reduced the clinical scores of EAE mice and drove the expansion of CNS Treg cells. The E3 ubiquitin ligase Nedd4 is involved in adaptive immune responses [54], and this protein regulates the stability of the Notch2 protein. Interestingly, the expression of Notch2 mRNA in infiltrated Notch2−expressing cells did not differ from that in CD4^+^Notch2^−^ cells. Notch2−expressing CD4^+^ T cells presented reduced expression of Nedd4, and forced expression of Nedd4 in cells resulted in downregulation of Notch2 expression, along with reduced expression of Foxp3, suggesting that Nedd4 regulates Notch2 expression by infiltrating CD4^+^ T cells. Because most CD4^+^Notch2^+^ cells express MOG−specific TCRs, repeated TCR stimulation may result in a dysfunctional phenotype and downregulation of Nedd4 expression in the cell population. In addition, the expression of Foxp3 through Notch2 signaling in the cell population plays an important role in the expansion of infiltrated Treg cells during remission from EAE. However, on the basis of previous studies in humans, the expression of PD−1 on pathogenic CD4^+^ T cells in chronic ulcerative colitis (UC) and inflammatory bowel disease (IBD) differs from that in other inflammatory diseases, including multiple sclerosis. Additionally, PBMCs from patients with severe UC and IBD contain a greater proportion of PD−1−expressing CD4^+^ T cells, and colonic tissue also presents elevated PD−1 expression [55–57]. Thus, in the chronic colitis state, PD−1 expression may not merely reflect CD4^+^ T cell exhaustion but may instead be associated with the long−term inflammatory nature of the disease.

Clinical trials investigating a TNF antagonist as a potential treatment for MS failed; rather, the antagonist exacerbated the clinical symptoms of MS [58]. In an experimental model, TNF (especially membrane−bound TNF) activated TNFR2 on Treg cells, thereby expanding the Treg cell population [59, 60]. By binding to and activating two distinct receptors, TNF-α is involved in diverse bioactivities: TNFR1 and TNFR2 [61]. TNFR1 is expressed ubiquitously, has conserved death domain motifs, and is activated by either soluble or membrane−bound TNF. The expression of TNFR2 is restricted to specific cell types, such as neurons, immune cells, and endothelial cells [60]. TNFR2 lacks a death domain and is therefore unable to induce programmed cell death directly; this receptor is suggested to be activated primarily by membrane−bound TNF [59]. One model of TNFR signaling proposes that TNFR1 signaling primarily promotes inflammation and tissue degeneration, whereas that of TNFR2 mediates local homeostatic effects such as cell survival and tissue regeneration [61]. This model suggests that selective therapeutic blockade of TNFR1 expression maintains homeostatic TNFR2 signaling; indeed, such a strategy is under development for the treatment of TNF−mediated diseases. Additionally, human peripheral blood CD4^+^CD25^+^Foxp3^+^ Treg cells express membrane−bound TNF, a potent activator of type 2 TNFR [62]. Surprisingly, CD4^+^Notch2^+^Foxp3^lo^ T cells expressed more membrane−bound TNF, and blocking TNF activity abolished population−mediated Treg expansion. Membrane−bound TNFα and TGF−β are specifically induced by the co−expression of Notch2 and Foxp3, whereas cells expressing only Foxp3 (Tregs) or only Notch2 without Foxp3 (CD4^+^Notch2^+^Foxp3^−^ T cells) do not exhibit elevated levels of these factors. Consistent with this, siRNA−mediated Notch2 inhibition disrupts Foxp3 expression, leading to reduced membrane−bound TNFα and TGF−β expression. Furthermore, our data clearly demonstrated direct interactions between CD4^+^Notch2^+^Foxp3^lo^ T cells and Treg cells. In addition, Foxp3−specific deletion of Notch2 reduces the number of proliferating Treg cells in the CNS of EAE mice, and animals fail to recover from EAE symptoms. Overall, the identified functions of CD4^+^Notch2^+^Foxp3^lo^ cells are consistent with previously reported results, including the failure of TNF antagonists to successfully treat MS and increased numbers of CNS−infiltrated Treg cells during the remission phase of EAE.

In conclusion, our results show that a previously unidentified CD4^+^Notch2^+^Foxp3^lo^ T cell population present in the spinal cords of EAE mice regulates EAE symptoms by driving expansion of the Treg cell population during the remission phase. Moreover, CD4^+^Notch2^+^Foxp3^lo^ T cells are present in the laminar propria of mice with experimental colitis and in that of humans with UC, whose population is phenotypically similar to that observed in an EAE mouse model. Thus, the expression of Notch2 could increase the expression of Foxp3 via CD4^+^ T cells with the dysfunctional phenotype, which then facilitates Treg cell expansion. Thus, CD4^+^Notch2^+^Foxp3^lo^ T cells function as immunoregulatory CD4^+^ T cells that play a central role in recovery from inflammation during autoimmunity. These findings suggest that CD4^+^Notch2^+^Foxp3^lo^ T cells may serve as novel mediators of the transition from the dysfunctional phenotype to immune regulatory conditions through Notch2 expression, potentially representing a new therapeutic target for autoimmune diseases.

## Materials and methods

### Mice and cells

CD45.1 ^+^ congenic mice (B6. SJL −*Ptprc*^*a*^ *Pepc*^*b*^*/BoyJ*), *Foxp3*^YFP − Cre^ (B6.129(Cg) −* Foxp3*^*tm4*(YFP/icre)Ayr^/J), *Foxp3*^eGFP^ (B6. Cg −* Foxp3*^tm1Mal^/J), and *Notch2*^flox/flox^ mice (B6.129S −* Notch2*^tm3Grid^/J) were purchased from Jackson Laboratory (Bar Harbor, Maine, USA). All the mice were kept under pathogen − free conditions in the Laboratory Animal Resource Center at the Gwangju Institute of Science and Technology and the Animal Center for Pharmaceutical Research at Seoul National University. *Notch2*^flox/flox^ mice were bred with *Foxp3*^YFP − Cre^ transgenic mice, and *Foxp3*^YFP − Cre^:*Notch2*^flox/flox^ mice were bred with *Foxp3*^YFP − Cre^:*Notch2*^flox/wt^ mice to generate *Foxp3*^YFP − Cre^:*Notch2*^flox/flox^ and *Foxp3*^YFP − Cre^:*Notch2*^flox/wt^ mice, respectively. All the mice used in the experiments were aged 6−10 weeks and were used in accordance with protocols approved by the Animal Care and Ethics Committees of Seoul National University. Mouse primary CD4^+^ T cells were isolated from the spinal cords, spleens, and inguinal lymph nodes of the mice via a FACSAria III Cell Sorter (BD Biosciences, Franklin Lakes, New Jersey, USA) or an EasySep Mouse CD4^+^ T cell isolation kit (Stem Cell, Vancouver, Canada). All animal experiments were approved by the Institutional Animal Care and Use Committee at Seoul National University and the Gwangju Institute of Science and Technology.

### Study design and participants

The study was approved by the Seoul National University Hospital’s Institutional Review Board (protocol number H−2012−115−1183). All patients provided informed written consent. The ages, sexes, and disease characteristics of all participants are provided in Supplementary Fig. 13A.

### Antibodies and proteins

Anti−mouse CD3 (17A2) and anti−mouse CD28 (145−2C11) antibodies were purchased from Bio X Cell (Seoul, Korea). The following were purchased from eBioscience (San Diego, California, USA): FITC−conjugated anti−mouse TCRβ (H57−597); Alexa Fluor 488−conjugated anti−mouse Foxp3 (FJK−16s), anti−human CD4 (OTK4), anti−human GITR (eBioAITR), and anti−human PD−1 (MIH4); PE−conjugated anti−human/mouse Notch2 (16F11) and anti−mouse CD25 (PC61.5) and CD69 (H1.2F3); Peridinin chlorophyll protein cyanine 5.5 (PerCP−Cy5.5)−conjugated anti−mouse CD3ε (145 − 2C11), IFN−γ (XMG1.2), CD62L (MEL−14), CD45.2 (104), and anti−human Foxp3 (RCH101); Allphycocyanin (APC)−conjugated anti−mouse Notch1 (22E5), anti−mouse/rat IL−17a (ebio17B7), anti−mouse CTLA4 (UC10−4B9) and ICOS (7E.17G9); anti−human/mouse CD44 (IM7) and CD45.1 (A20), and anti−human/mouse CTLA4 (UC10−4B9); PE−cy7−conjugated anti−mouse GITR (DTA−1), OX40 (OX−86), PD−1 (J43), CD4 (GK 1.5), and anti−human/mouse LAP (TW7−16B4); efluor450−conjugated anti−mouse CD4 (GK 1.5) and anti−human/mouse TNFα (MP6−XT22); and Brilliant Ultraviolet 737−conjugated anti−human CD8a (RPA−T8). The following antibodies were purchased from BioLegend (San Diego, California, USA): PE−conjugated anti−mouse Tim3 (5D12), Brilliant Ultraviolet 395−conjugated anti−human CD3ε (UCHT1), and PerCP−Cy5.5−conjugated anti−mouse Lag3 (C9B7W). The following antibodies were purchased from BD Pharmingen: APC−conjugated anti−mouse Notch2 (HMN2−35); Brilliant Violet 785−conjugated anti−human PD−1 (NAT105); Brilliant Violet 605−conjugated anti−human CD336 (Tim3, F38−2E2); Brilliant Violet 711−conjugated anti−human CD223 (Lag3, 11C3C65); and Alexa Fluor 700−conjugated anti−human CD4 (RM4−5). The following antibodies were purchased from Santa Cruz (Dallas, Texas, USA): Alexa 488−conjugated anti−mouse/rat/human Jag1 (E−12).

### Isolation of mononuclear leukocytes (MNLs) from the spinal cords of EAE mice

Spinal cords were isolated from EAE mice after perfusion with PBS. Each spinal cord sample was chopped in PBS containing collagenase D (2.5 mg/ml; Sigma‒Aldrich, Burlington, USA) and DNase I (1 mg/ml; Roche, Grenzacherstrasse, Basel, Switzerland) and then incubated in a 37 °C shaking incubator for 1 h. After incubation, spinal cord homogenates were produced by passing the tissue (in PBS) through a 70 μm nylon strainer, followed by centrifugation. The supernatants were discarded, and the remaining cells were purified on a Percoll (GE Healthcare, Chicago, Illinois, USA) gradient. Briefly, the cell pellets were suspended in 4 ml of 37% (v/v) Percoll, and a layer was carefully formed beneath the mixture using 4 ml of 70% (v/v) Percoll. This sample was then centrifuged at 1500 × *g* for 30 min with no brake. After centrifugation, the layer containing the cells was harvested and washed with PBS.

### Isolation of MNLs from the colons of colitis model mice

The colons were removed from the mice with DSS−induced colitis, chopped in 1× HBSS containing 5 mM EDTA and 1 mM DTT, and then incubated for 30 min in a 37 °C shaking incubator. After incubation, the tissues (in 1× HBSS) were passed through a 100 μm nylon strainer, and this process was repeated twice. The remaining colon pieces were incubated in 1× PBS containing collagenase D, DNase I, and dispase II, followed by incubation for 30 min in a 37 °C shaking incubator. After incubation, the homogenates were produced by passing the tissue (in 1× PBS) through a 70 μm nylon strainer, and this process was repeated twice. After centrifugation, the supernatants were discarded, and the remaining cells were purified on a Percoll gradient. Briefly, the cell pellets were suspended in 40% (v/v) Percoll. Next, 4 ml of 80% (v/v) Percoll was carefully layered beneath this solution. The sample was then centrifuged at 1500 × *g* for 30 min with no brake. After centrifugation, the layer containing the cells was harvested and washed with PBS.

### Isolation of MNLs from the colon of IBD patients

Colon biopsies in 1× PBS were immediately transported to the laboratory. Next, the tissues were chopped in 1× PBS containing collagenase D (2.5 mg/ml; Sigma‒Aldrich, Burlington, St. Louis, USA) and DNase I (1 mg/ml; Roche), followed by incubation for 30 min at 37 °C in a shaking incubator. After incubation, the tissues (in 1× PBS) were passed through a 100 μm nylon strainer, and this process was repeated twice. After centrifugation, the supernatants were discarded, and the remaining cells were purified on a Percoll gradient as described in the previous section.

### siRNA electroporation

Notch2 siRNA (1320001) was purchased from Thermo Fisher Scientific (Waltham, Massachusetts, USA). The control siRNA (ss−1011) was purchased from Bioneer (Daejeon, Korea). The siRNA (1 μM) was electroporated via a 4D−Nucleofector (Lonza, Muenchensteinerstrasse, Basel, Switzerland). After 48 h, the electroporated cells were harvested and analyzed via flow cytometry.

### MOG_38 − 49 _−IA^b^ tetramer staining

MOG_38 − 49 _−IA^b^ monomers were obtained from the NIH Tetramer Facility. PE−conjugated streptavidin was purchased from BioLegend. The tetramers were formed by individually incubating class II molecules with labeled streptavidin for 12–18 h at room temperature at a molar ratio of 8:1. PE−conjugated MOG_38 − 49 _−IA^b^ tetramer staining was performed at RT for 30 min.

### Confocal immunofluorescence analysis

The samples were fixed in 10% formalin in PBS and then washed with 1× PBS. The samples were then placed in 1× PBS containing 30% sucrose and incubated overnight at 4 °C. Next, the samples were embedded in either optimal cutting temperature compound or formalin−fixed paraffin−embedded (FFPE) blocks. FFPE blocks were subjected to antigen retrieval and permeabilization with Triton X−100. After sample preparation, images were acquired via a TCS8 (Leica, Wetzlar, Germany) confocal microscope or a superresolution confocal microscope (LSM900, ZEISS, Oberkochen, Germany).

### Confocal live and 3D imaging analysis

Labeled cells were cultured in cover glass−bottom confocal dishes (SPL, Gyeonggi−do, Korea) coated with anti−CD3ε (2 μg/ml) and anti−CD28 (2 μg/ml) antibodies. After 1 day, the confocal dish was placed on a confocal scope TCS8 stage under temperature−, CO_2-_, and humidity−controlled conditions. For 3D imaging, a 4% formaldehyde−fixed plate was placed on a superresolution LSM900 confocal microscope.

### Quantitative RT‒PCR

Total RNA was extracted from naïve CD4^+^ T cells and infiltrated CD4^+^ T cells isolated from the spleens and spinal cords of EAE mice via a Qiagen RNeasy Micro Kit (QIAGEN, Hilden, Germany). Complementary DNAs were generated via TOPscript RT DryMIX (Daejeon, Korea). The primers used were designed via PrimerBank (https://pga.mgh.harvard.edu/primerbank/) and synthesized by Macrogen (Seoul, Korea). Quantitative RT‒PCR was performed using SYBR Green Master Mix (Enzynomics, Daejeon, Korea) and an Mx3005p quantitative PCR system (Stratagene, La Jolla, California, USA). Glyceraldehyde−3−phosphate dehydrogenase or β−actin served as internal controls. Analysis was performed via the 2−ΔΔCT method, except for Hes5 mRNA (which was not detected in control cells), for which the 2−ΔCT method was employed.

### Flow cytometry analysis

MNLs were isolated from the spinal cord, splenocytes, and lymphocytes of 6– to 10−week−old mice. To detect cell surface antigens, the cells (2 × 10^5^) were labeled with fluorochrome−conjugated primary antibodies (30 min at RT). To identify the IFN−γ, IL−17a, and Foxp3−expressing cell populations, isolated CD4^+^ T cells (2 × 10^5^) were cultured for 4 h with phorbol myristate acetate (PMA; 50 ng/ml), ionomycin (1 μg/ml), and brefeldin A (1×). To detect intracellular antigens, surface−stained cells were fixed and permeabilized with permeabilization buffer (eBioscience) or Foxp3/Transcription Factor Staining Buffer (eBioscience). The stained cells were analyzed on a FACSCanto II instrument (BD Biosciences) or a high−end performance flow cytometer (LSR Fortessa X−20; BD Biosciences).

### EAE model and adoptive transfer

Mice (8–10 weeks old) were immunized subcutaneously (s.c.) with 200 μg of MOG p35−55 peptide (Peptron, Daejeon, Korea) mixed with supplemented complete Freund’s adjuvant (Sigma‒Aldrich), followed by intraperitoneal (i.p.) injection of *Bordetella pertussis* toxin (400 ng per mouse; List Biological Laboratories, Campbell, California, USA) on Days 0 and 2. Clinical EAE was graded on a scale of 1−5 on the basis of established criteria54: 0, no observable symptoms; 1, tail paralysis; 2, partial hind limb weakness; 3, hind limb paralysis; 4, hind paralysis and forelimb weakness; and 5, moribund or dead. For adoptive transfer, spinal cord−infiltrated CD4^+^ T cells were isolated from EAE−induced *Foxp3*^eGFP^ mice (CD45.2) as previously described. Spinal cord lymphocytes were sorted on the basis of the expression of Notch2 and Foxp3 (i.e., CD4^+^Notch2^+^ T cells and CD4^+^Notch2^+^Foxp3^lo^ T cells) via a FACS Aria III Cell Sorter (BD Biosciences). At 10−12 days post−EAE induction, the mice (CD45.1) were injected with PBS, CD45.2^+^CD4^+^Notch2^−^ T cells, or CD45.2^+^ CD4^+^Notch2^+^Foxp3^lo^ T cells. The number of cells transferred through the retro-orbital vein was 5 × 10^5^ per mouse. Disease severity was scored on a scale of 0 to 5 (0, no disease; 1, loss of tail tone; 2, hind limb weakness; 3, hind limb paralysis; 4, hind limb paralysis and forelimb paralysis or weakness; and 5, moribund/death). The infiltrating Th population was counted via FACS analysis, cytokine production was measured (see below), and histological analysis was performed.

### DSS model

The mice (8–10 weeks old) were exposed to 2.5% DSS in the drinking water for 5 days, followed by access to regular drinking water until the end of the experiment. Body weight was scored daily.

### Cytokine analysis

To measure the levels of secreted cytokines, CD4^+^ T cells were isolated from the spinal cords of EAE mice. Isolated CD4^+^ T cells (2 × 10^5^) were cultured for 6 h with PMA (50 ng/ml) and ionomycin (1 μg/ml). Secreted IFN−γ, IL−17a, IL−2, IL−6, and TGF−β levels were measured via a BD Cytometric Bead Array (BD Biosciences). The levels of intracellularly stained cytokines were analyzed via a FACSCanto II or LSRFortessa flow cytometer (BD Biosciences).

### Proliferation assay

To analyze the proliferation of activated cells, a CellTrace Violet (CTV) or CFSE Cell Proliferation Kit (Thermo Fisher Scientific) was used. CD4^+^ T cells were isolated from the spleens and spinal cords of EAE mice, and the isolated cells were stained with CTV (5 μM). To analyze the suppressive effect on proliferation, CTV−stained CD4^+^ T cells (1 × 10^5^) were cocultured at a 1:1 ratio with CD4^+^Notch2^−^ T cells, CD4^+^Notch2^+^Foxp3^−^ T cells, CD4^+^Notch2^+^Foxp3^lo^ T cells, or Treg cells. Activation was performed via anti−CD3/CD28 Dynabeads at a 1:1 bead-to-CD4^+^CD25^−^ T cell ratio. Treg cells were sorted by using CD25 and Foxp3−eGFP. Treg cell expansion was analyzed by CTV or CFSE under activation conditions, and Treg cells were cocultured with CD4^+^Notch2^−^ T cells, CD4^+^Notch2^+^Foxp3^−^ T cells, and CD4^+^Notch2^+^Foxp3^lo^ T cells at a 1:1−to−3:1 ratio and activated with anti−mouse CD3ε (2 μg/ml) and anti−mouse CD28 (2 μg/ml) antibodies for 72 h. The intensity of CTV on the cells was measured via a FACSCanto II flow cytometer (BD Biosciences) or an LSRFortessa flow cytometer (BD Biosciences).

### iTreg cell differentiation with CD4^+^ T cells infiltrating the CNS in EAE

CD4^+^ T cells isolated from the spleen were activated with anti−CD3ε (2 μg/ml) and anti−mouse CD28 (2 μg/ml) antibodies. Under these conditions, CD4^+^ T cells were cocultured for 72 h at a 1:1 ratio with CD4^+^ Notch2^−^ T cells, CD4^+^Notch2^+^Foxp3^−^ T cells, CD4^+^Notch2^+^Foxp3^lo^ T cells, and Tregs, which were isolated from the CNS of EAE patients. Foxp3 expression was measured via a FACSCanto II flow cytometer (BD Biosciences) or an LSRFortessa flow cytometer (BD Biosciences).

### CRISPR/Cas9 gene editing of *NEDD4*

Two single−guide RNAs (sgRNAs) were used to target mouse *Nedd4*: crRNA1, ATGGGTATGGGAGTTTTGCC; crRNA2, CACCATCTTCTGTCTTATCC (PhileKorea, Inc.). Cas9−GFP (Merk) and sgRNAs were incubated at room temperature for 10 min in a 3:1 reaction. A total of 1 × 10^6^ cells were electroporated with the RNP complex. The KO efficiency of the genes was confirmed via genomic PCR and the TIDE assay. PCR was performed via the following primers: TCCACAGCTTGGAGACATTACC forward primer, GTAACATCACTTCCGGGGGA reverse primer (Macrogen) with the Q5 Hot Start High−Fidelity 2X Master Mix (New England Biolabs).

### Abseq surface labeling and single − cell RNA − sequencing analysis

Isolated spinal cord mononuclear cells (1 × 10^6^) were labeled with mouse CD4 Olgo AMM2002 (RM4−5) and mouse Notch2 Olgo AMM2129 (16F11) for 30 min at 4 °C and washed three times before the experiments were performed. The cells were resuspended in BD Rhapsody Cartridge reagent kit sample buffer, and 4 × 10^4^ cells were captured with the BD Rhapsody single−cell system following the manufacturer’s instructions. Additionally, antibody tag libraries, multiplexing libraries and targeted mRNA gene expression libraries were generated following the manufacturer’s instructions. Single−cell transcriptomics analysis was performed via the Python package Scanpy (v.1.9.1). Cell clustering and UMAP visualization were performed on statistically significant principal components.

### Quality control and data processing

Cells with fewer than 500 unique transcripts and more than 10% mitochondrial gene content were excluded. Additionally, cells displaying a unique gene counting over 7500 were considered outliers and removed, resulting in a final dataset of 16,417 cells after quality control. The count data were log − normalized using pp.normalize, and highly variable genes were selected with the pp.highly_variable_gene function (flavor = “seurat_v3”, incorporating 2000 genes). Principal component analysis (PCA) was performed, and 30 components were retained for downstream analysis. To correct batch effects across samples, the Harmony algorithm was applied.

### CD4 ^+^ T-cell subset analysis

To investigate CD4^+^ T cell subsets, we subset the CD4^+^ T cell clusters from the dataset for focused analysis. Using Leiden clustering (resolution = 1.0), we identified 10 distinct clusters, each annotated on the basis of a unique gene marker profile. This classification enabled the categorization of CD4^+^ T cells into eight subclusters, providing deeper insights into their functional diversity.

### scFates for trajectory analysis

For trajectory inference within CD4^+^ T cells, we first computed a PCA representation of the data matrix using sc.pp.neighbors. A neighborhood graph was then embedded with UMAP after computing the diffusion maps (sc.tl.diffmap). By identifying key transition nodes between clusters, we reconstructed six major trajectories within the CD4^+^ T cell subpopulations. This analysis delineated distinct transcriptional pathways and molecular signatures associated with each trajectory, offering insights into CD4^+^ T cell differentiation and functional specialization.

### Statistical analysis

Statistical analyses of the differences between two groups were performed via Origin2021 software (OriginLab, Northampton, Massachusetts, USA) and Graph Prism v.10.0.3 (GraphPad, Boston, Massachusetts, USA). The statistical tests used to determine significance in each analysis are described in the respective figure legends of the corresponding figures. The results are displayed as the means ± SDs. Significance is indicated as *p < 0.05, **p < 0.01, and ***p < 0.001.

## Supplementary information

Supplementary Materials without Highlight

Treg and CD4^+^Notch2^+^Foxp3^lo^ T cells interaction

Source for Fig. 6D

Source for Fig. 6G

## Data Availability

All the data reported in this paper will be shared by the lead contact upon request.
