# Supplementary material for: Notch2−expressing CD4+ T cells attain immunoregulatory functions during autoimmune inflammation
Source: Cell Mol Immunol. 2025 Jul 23;22(9):1077–92. doi: 10.1038/s41423-025-01318-2 (PMC12398495; doi:10.1038/s41423-025-01318-2)
Supplement: Supplementary file 1 — Supplementary Materials without Highlight [file 41423_2025_1318_MOESM1_ESM.docx]

**Supplementary Materials for**

**Notch2-expressing CD4^+^ T cells attain immunoregulatory functions during autoimmune inflammation**

So−Eun Bae^1, 8^, Sang−Heon Park^1, 8^, Chae Youn Kim^2^, Cho−Rong Lee^1^, Chanyeon Lee^1^, Rosah May Payumo^4, 5^, So Yeon Kim^5, 6^, Kyu−Young Sim^1^, Ho Jin Kim^6^, Hyungseok Seo^1^, Seong−Joon Koh^7^, Seunghee Hong^2, 3^, Sung−Gyoo Park^1✉^

^1^Institute of Pharmaceutical Sciences, College of Pharmacy, Seoul National University, Seoul 08826, Republic of Korea. ^2^Department of Biochemistry, College of Life Science and Biotechnology, Yonsei University, Seoul 03722, Republic of Korea. ^3^Brain Korea 21 (BK21) FOUR Program, Yonsei Education & Research Center for Biosystems, Yonsei University, Seoul 03722, Republic of Korea. ^4^National Cancer Center−Graduate School of Cancer Science and Policy, Goyang 10408, Republic of Korea. ^5^Division of Clinical Research, Research Institute, National Cancer Center, Goyang 10408, Republic of Korea. ^6^Department of Neurology, Hospital of National Cancer Center, Goyang 10408, Republic of Korea. ^7^Division of Gastroenterology and Hepatology, Department of Internal Medicine, College of Medicine, Seoul National University, Seoul 03080, Republic of Korea. ^8^These authors contributed equally: So−Eun Bae, Sang−Heon Park. ^✉^e−mail: riceo2@snu.ac.kr

**The PDF file includes:**

Supplementary Fig. 1 to 13

Supplementary Table 1

**Other Supplementary Materials for this manuscript include the following:**

Supplementary Video 1

**Supplementary Figures**


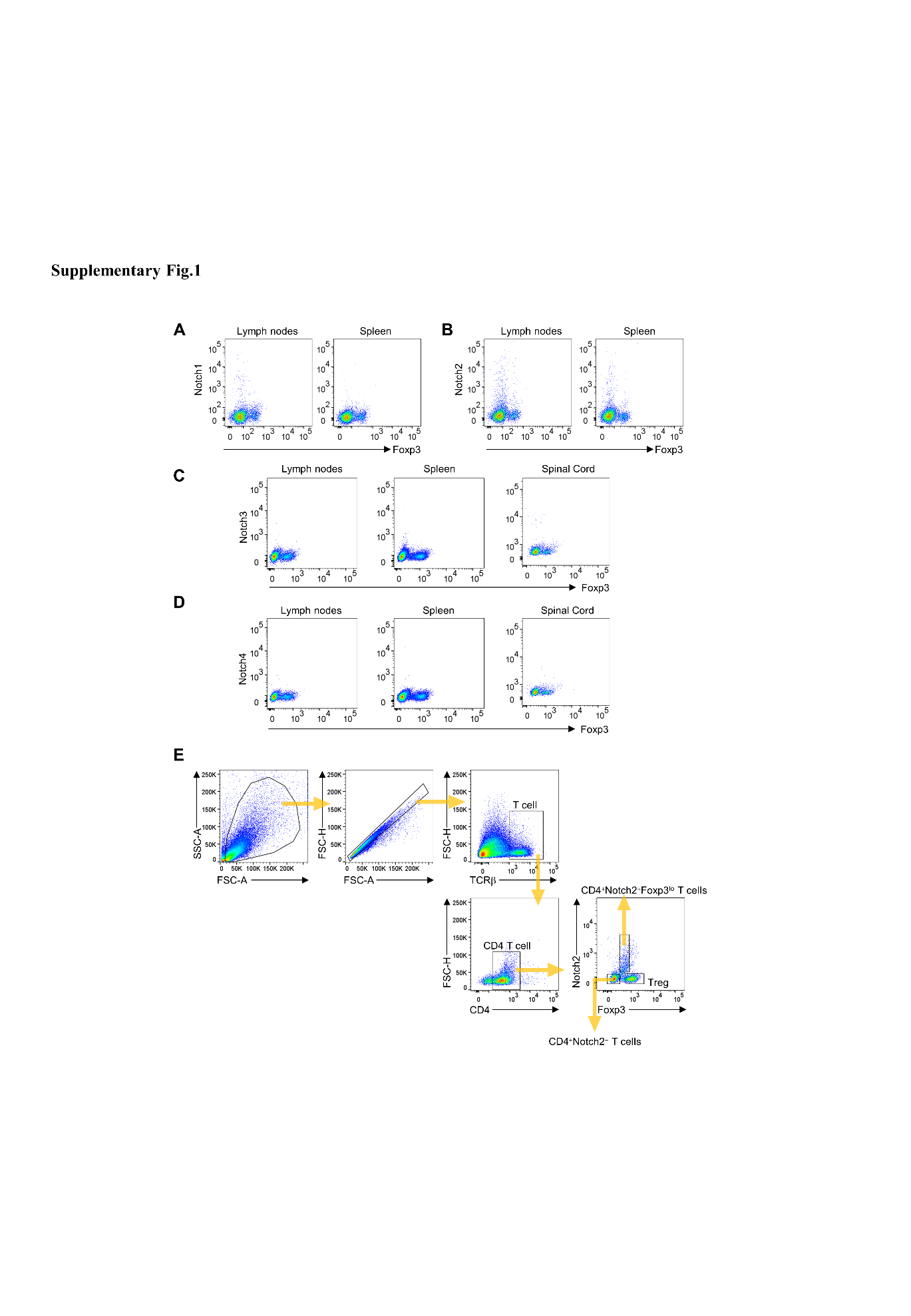


Supplementary Fig. 1. Analysis of Notch1−4 expression on CD4^+^ T cells in the peripheral lymphoid organs of EAE mice (related to Fig. 1) and sorting strategy. A Representative flow cytometry analysis of Notch1 and Foxp3 expression by CD4^+^ T cells from the lymph nodes and spleens of EAE model mice. B Representative flow cytometry analysis of Notch2 and Foxp3 expression by CD4^+^ T cells from the lymph nodes and spleens of EAE model mice. C Representative flow cytometry analysis of Notch3 and Foxp3 expression by CD4^+^ T cells from the lymph nodes, spleens, and spinal cord of EAE model mice. D Representative flow cytometry analysis of Notch4 and Foxp3 expression by CD4^+^ T cells from the lymph nodes, spleens, and spinal cord of EAE model mice. E Sorting gating strategy. Data are representative of three (A−D) independent experiments.


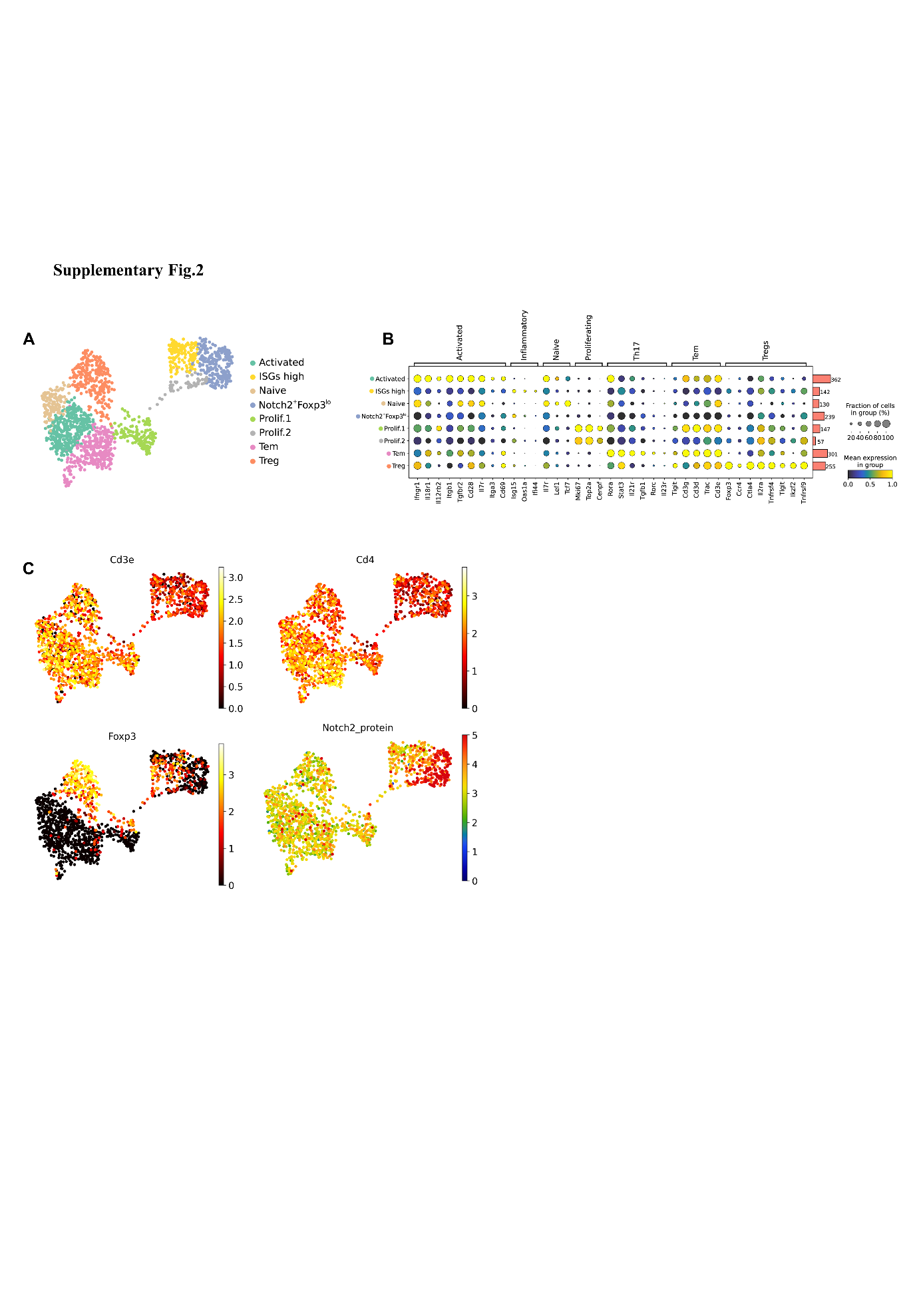
Supplementary Fig. 2. Single−cell sequencing analysis of CNS−infiltrated CD4^+^ T cells in EAE (related to Fig. 1). A UMAP plot depicting eight distinct subclusters of CD4^+^ T cells. B Cell−type annotation was performed using canonical marker genes, shown in the dot plot. Color intensity represents scaled mean expression, and dot size indicates the fraction of gene expression across clusters. The pink box plots on the right represent the number of cells in each cluster. C UMAP plots showing the expression of marker genes and Notch2 protein.


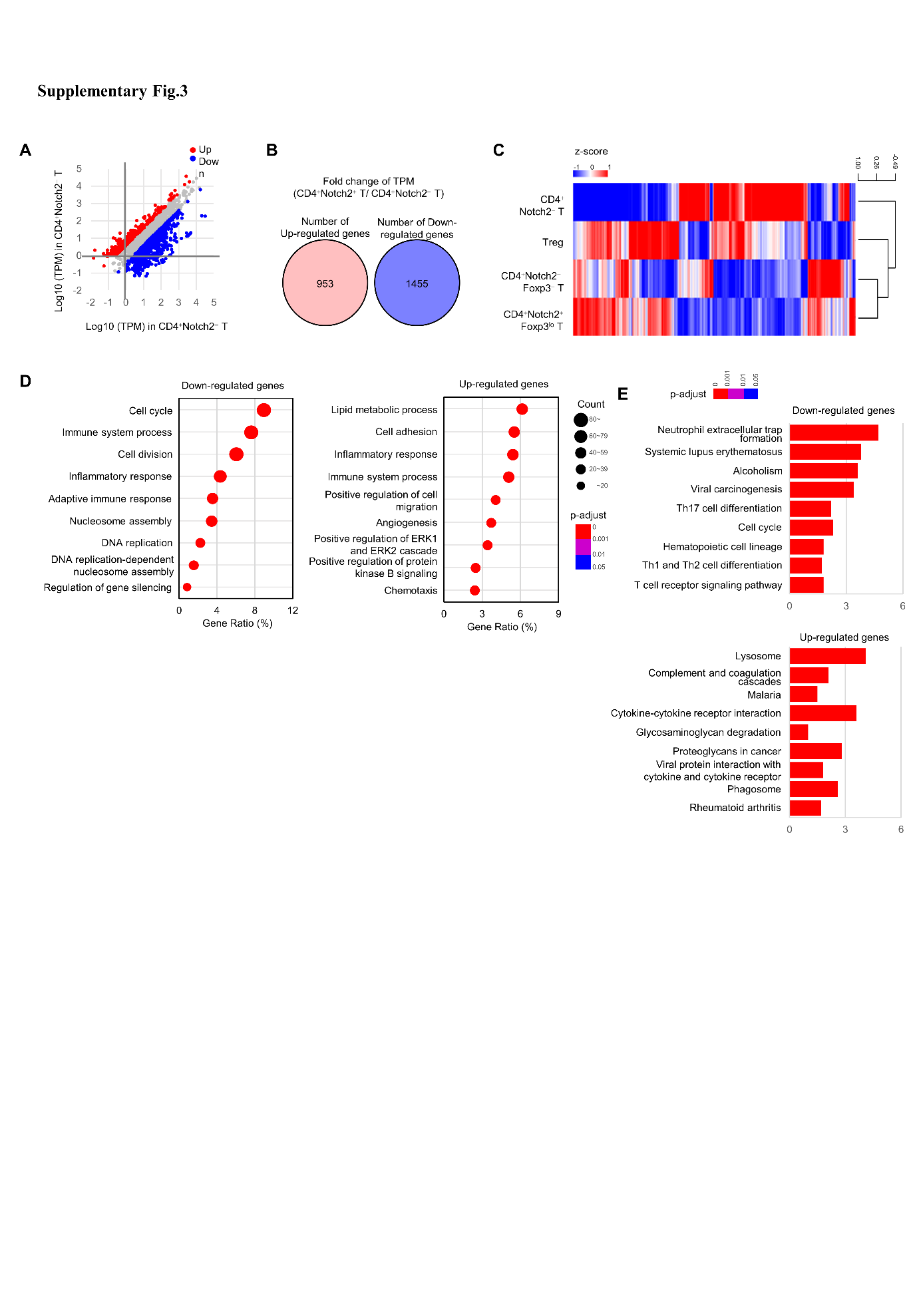
Supplementary Fig. 3. CNS−infiltrating CD4^+^Notch2^+^ T cells are transcriptionally different from infiltrating CD4^+^Notch2^−^ T cells (related to Fig. 2). A Scatter plots showing genes differentially expressed by CNS−infiltrated CD4^+^Notch2^+^ T cells and CD4^+^Notch2^−^ T cells (units = transcripts per million (TPM)). B Venn diagram showing the number of genes upregulated (pink, fold change ≥ 2) or downregulated (purple, fold change ≤ 0.5) based on TPM of CD4^+^Notch2^+^ T cells versus TPM of CD4^+^Notch2^−^ T cells. C Heatmap showing genes differentially expressed by CD4^+^Notch2^−^ T cells, Treg cells, CD4^+^Notch2^+^Foxp3^−^ T cells, and CD4^+^Notch2^+^Foxp3^lo^ T cells. D Gene Ontology (GO) biological process categories related to downregulated (upper panel, -fold change ≤ 0.5) and upregulated (lower panel, −fold change ≥ 2) genes (TPM) in CD4^+^Notch2^+^ T cells versus CD4^+^Notch2^−^ T cells. E Kyoto Encyclopedia of Genes and Genomes (KEGG) pathway categories related to downregulated (left panel, -fold change ≥ 2) and upregulated (right panel, −fold change ≤ 0.5) genes (TPM) in CD4^+^Notch2^+^ T cells versus CD4^+^Notch2^−^ T cells. All the GO and KEGG groups were identified according to the EASE scores (*P* ≤ 0.05).


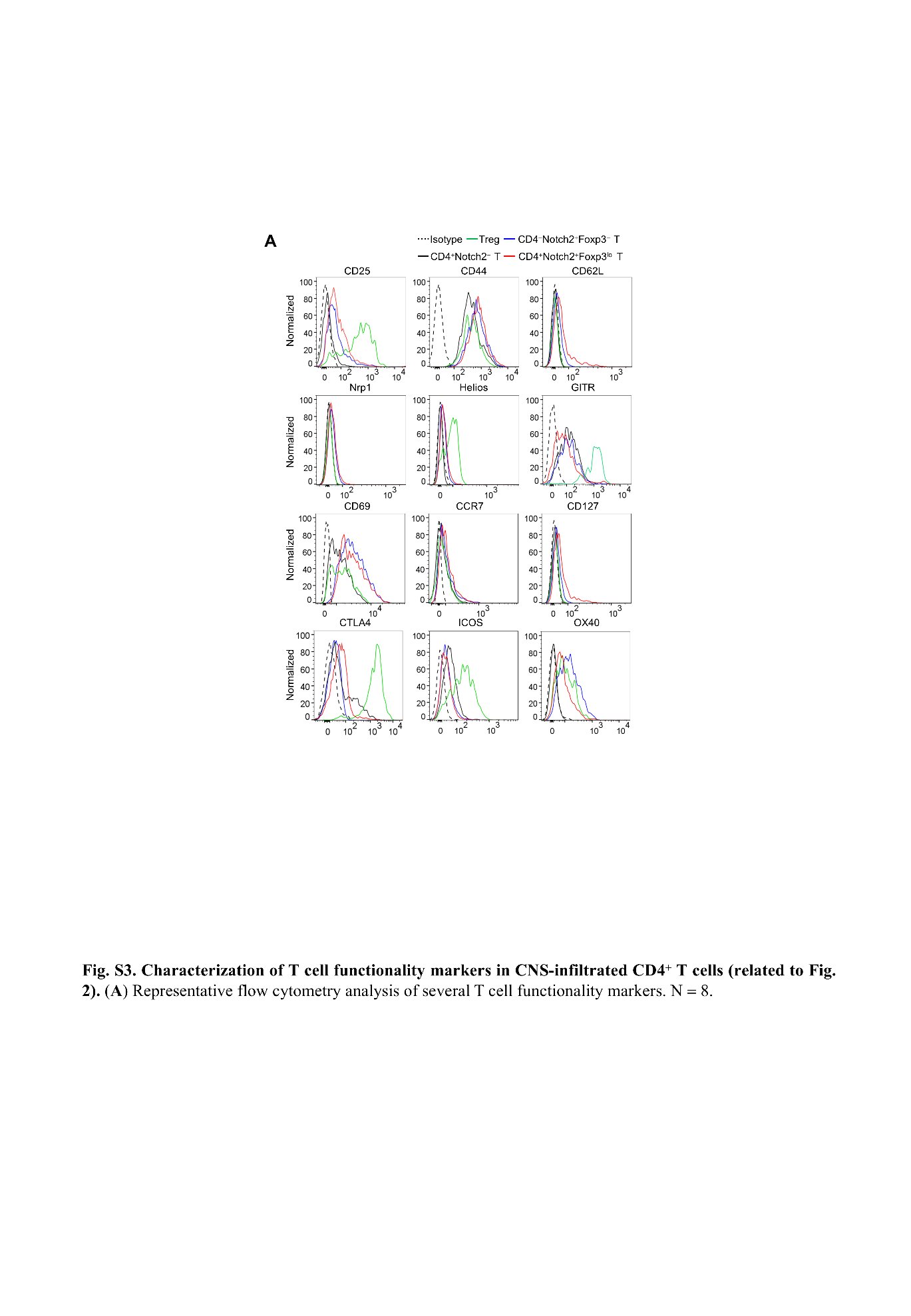


Supplementary Fig. 4. Characterization of T cell functionality markers in CNS−infiltrated CD4^+^ T cells (related to Fig. 2). A Representative flow cytometry analysis of several T cell functionality markers. Data are representative of three (A) independent experiments.


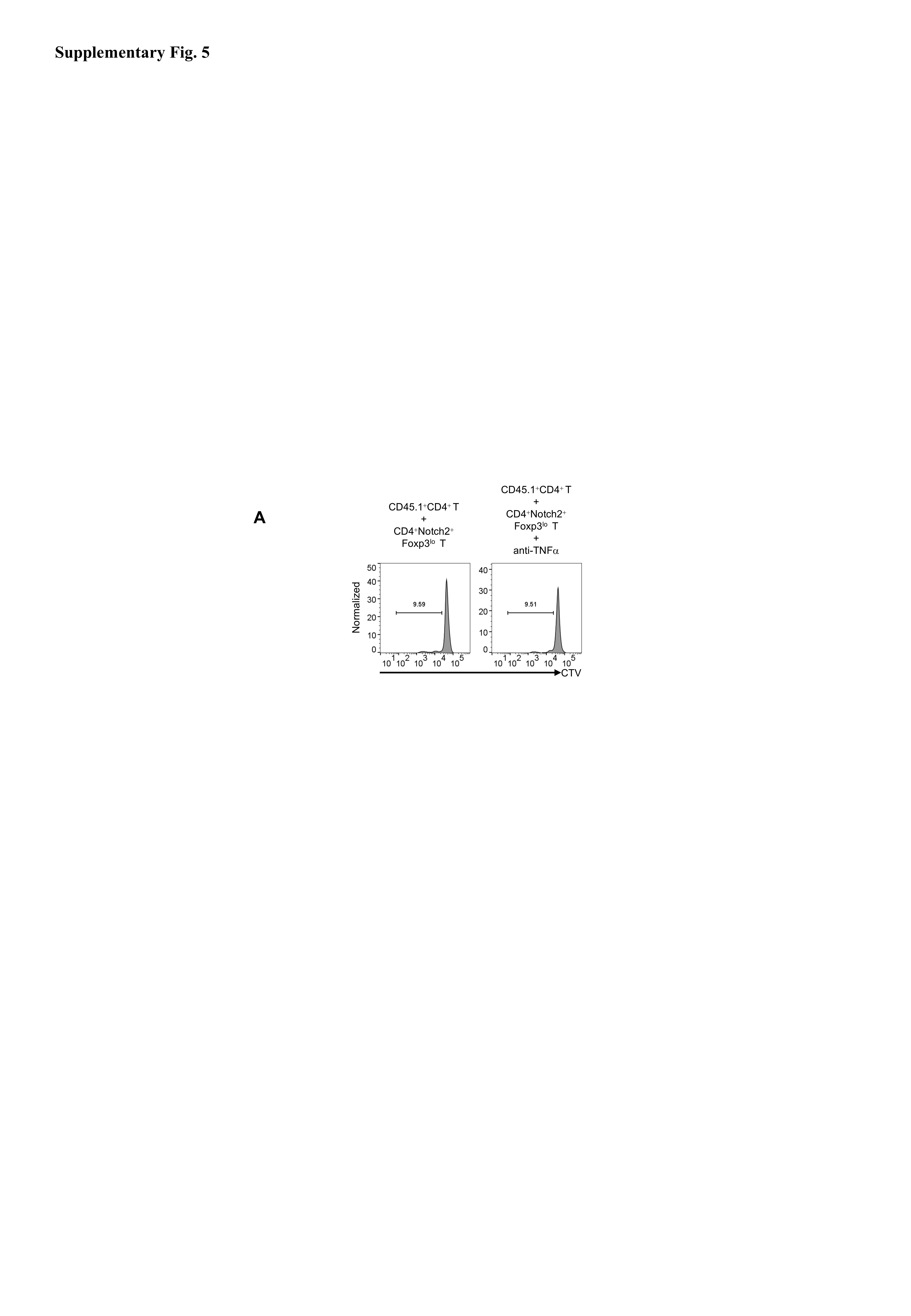


Supplementary Fig. 5. CD4^+^ T cells proliferation suppression assay (related to Fig. 2). A Representative suppression of CD4^+^ T cell proliferation by CD4^+^Notch2^+^Foxp3^lo^ with or without anti−TNFα antibodies. Data are representative of three independent experiments.


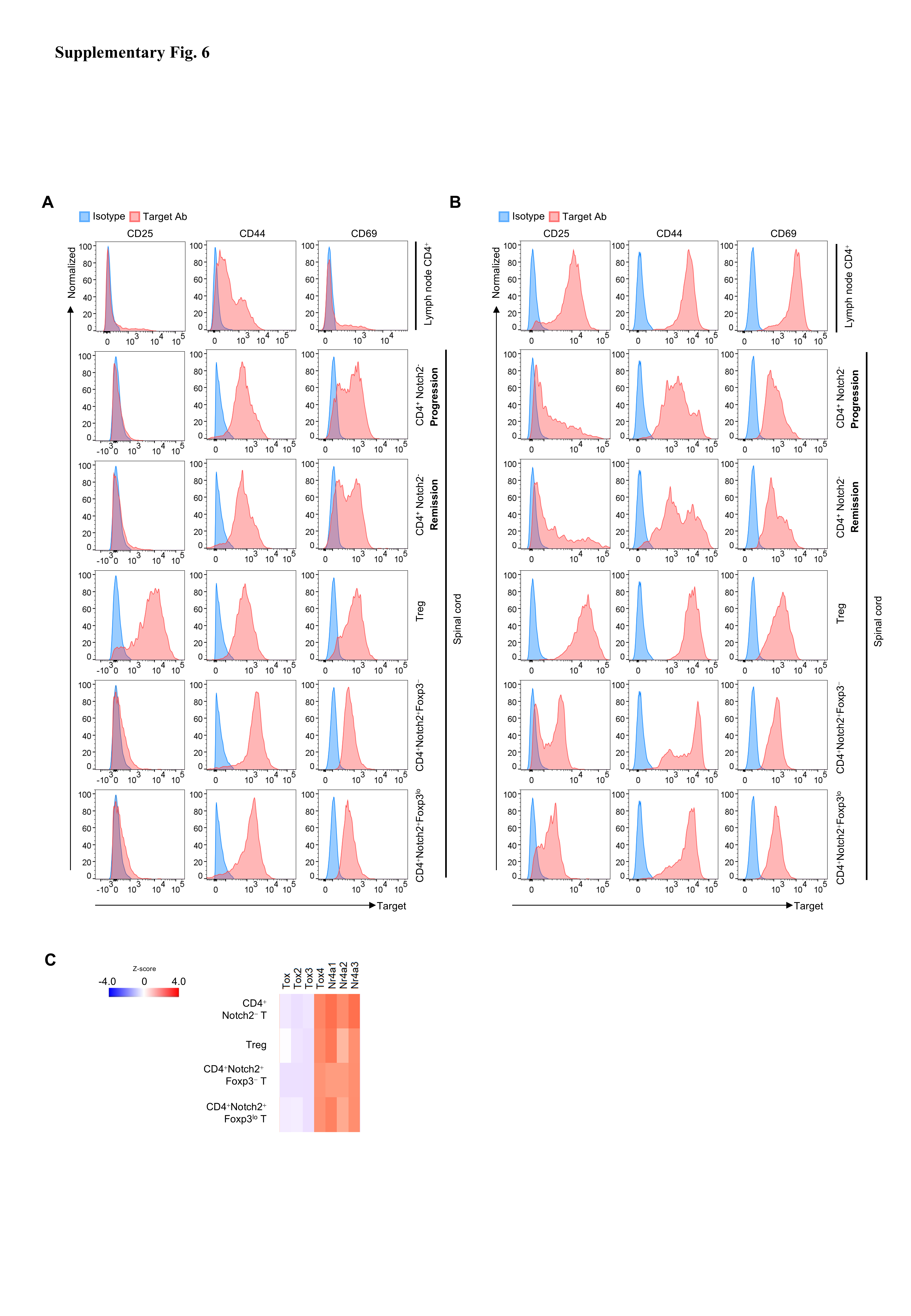


**Supplementary Fig. 6. Characterization of T cell activation markers and exhaustion−related genes in CNS−infiltrated CD4^+^ T cells (related to Fig. 2). A** Representative flow cytometry analysis of CD44, CD69, and CD25 expression in CD4^+^ T cells from lymph nodes and spinal cord of EAE mice. **B** Representative flow cytometry analysis of CD44, CD69, and CD25 expression upon TCR/CD28 stimulation. **C**. Heatmap showing expression of exhaustion−related transcripts. Data are representative of three independent experiments (**A**−**B**).


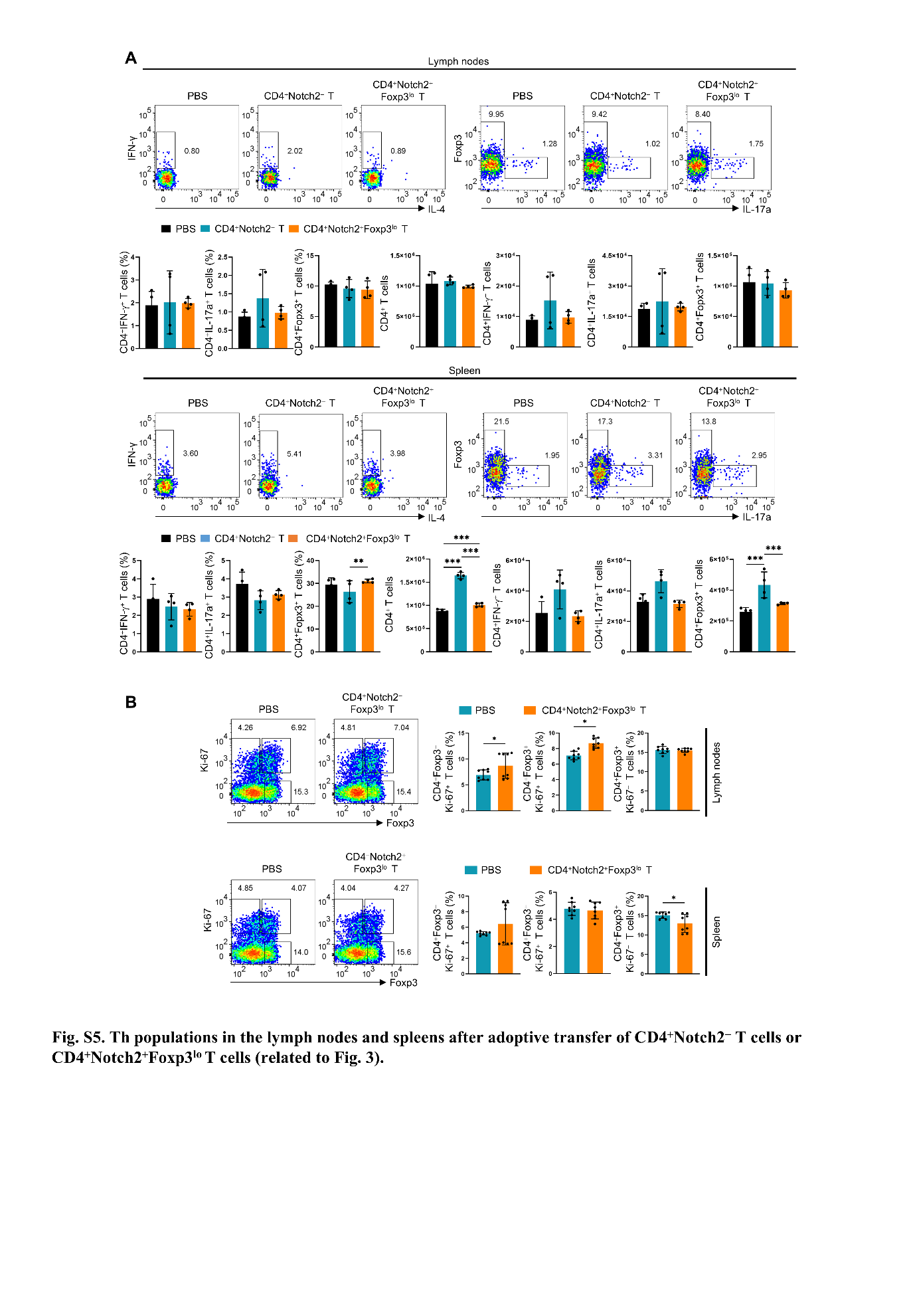
Supplementary Fig. 7. IL−17a^+^, IFN−γ^+^, IL−4^+^, and Foxp3^+^ T cells in the lymph nodes and spleens after adoptive transfer of CD4^+^Notch2^−^ T cells or CD4^+^Notch2^+^Foxp3^lo^ T cells (related to Fig. 3). A IL−17a^+^, IFN−γ^+^, IL−4^+^, and Foxp3^+^ T cells in the lymph nodes (upper panel) and spleens (bottom panel) of EAE model mice subjected to adoptive transfer. Graph shows the mean ± SDs of cell number and percentage. B Ki−67 and Foxp3 expression by CD4^+^ T cells in the lymph nodes (upper panel) and spleens (bottom panel) of mice subjected to adoptive transfer was analyzed via flow cytometry to detect proliferating Foxp3−expressing cells. Graph shows the mean ± SDs of cell percentage. Data are representative of three (A, B) independent experiments (error bars, s.d. of four (A) or eight (B) mice). Statistical significance was calculated via two−way ANOVA followed by Tukey’s multiple comparison in (A) and Sidak’s multiple comparison in (B); **P* < 0.05, ***P* < 0.01, ****P* < 0.001.


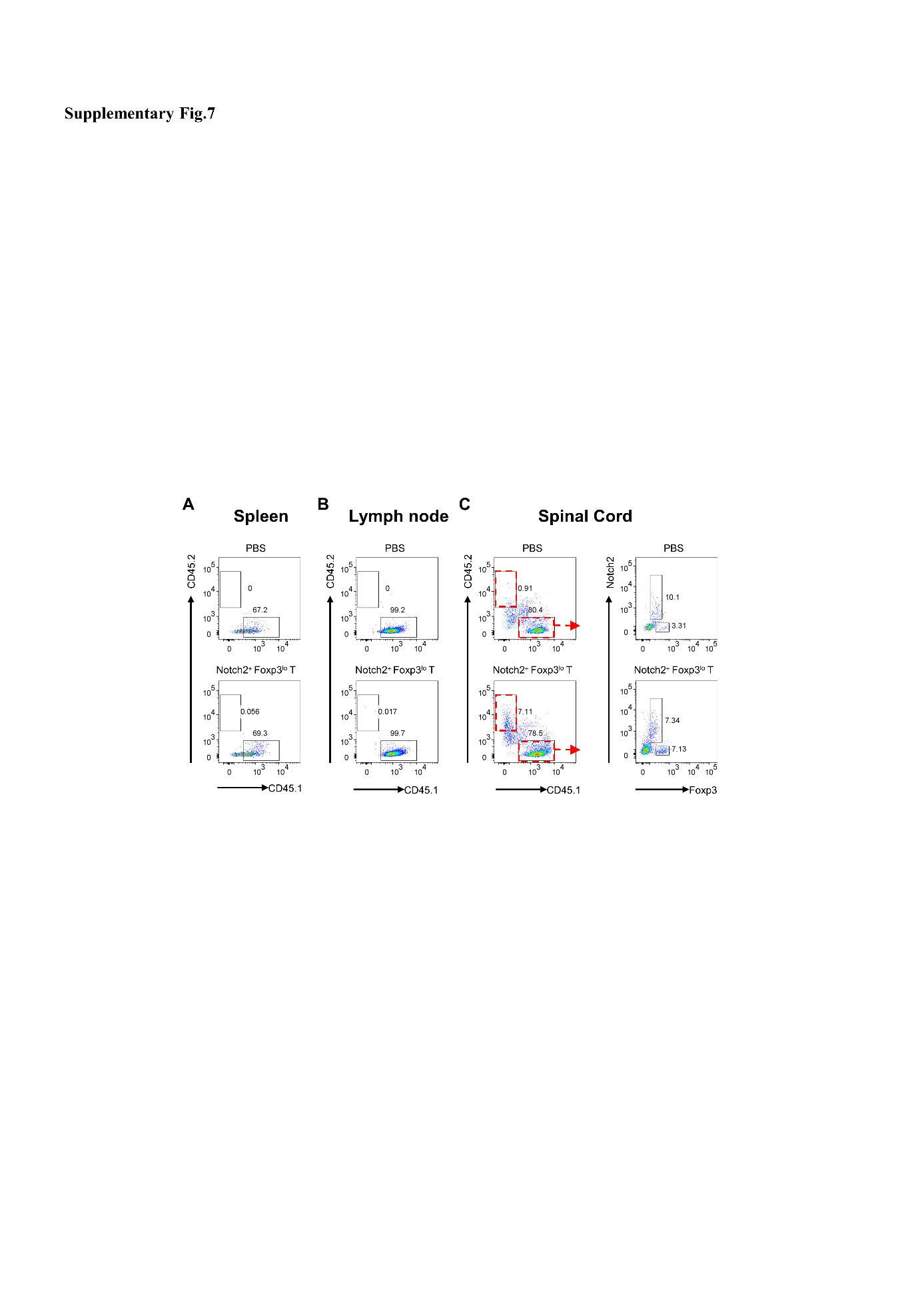


Supplementary Fig. 8. Infiltration of adoptively transferred CD4^+^Notch2^+^Foxp3ˡᵒ T cells into the spleen, lymph nodes, and spinal cord (related to Fig. 3). A Representative flow cytometry analysis of adoptively transferred CD45.2^+^CD4^+^ T cells and recipient−derived CD45.1^+^CD4^+^ T cells among total CD4^+^ T cells in the spleen of EAE mice. B Representative flow cytometry analysis of adoptively transferred CD45.2^+^CD4^+^ T cells and recipient−derived CD45.1^+^CD4^+^ T cells among total CD4^+^ T cells in the lymph node of EAE mice. C Representative flow cytometry analysis of adoptively transferred CD45.2^+^CD4⁺ T cells and recipient−derived CD45.1^+^CD4^+^ T cells among total CD4^+^ T cells in the spinal code of EAE mice. Notch2 and Foxp3 expression in recipient-derived CD45.1^+^CD4^+^ T cells. Data are representative of four (A−C) independent experiments.


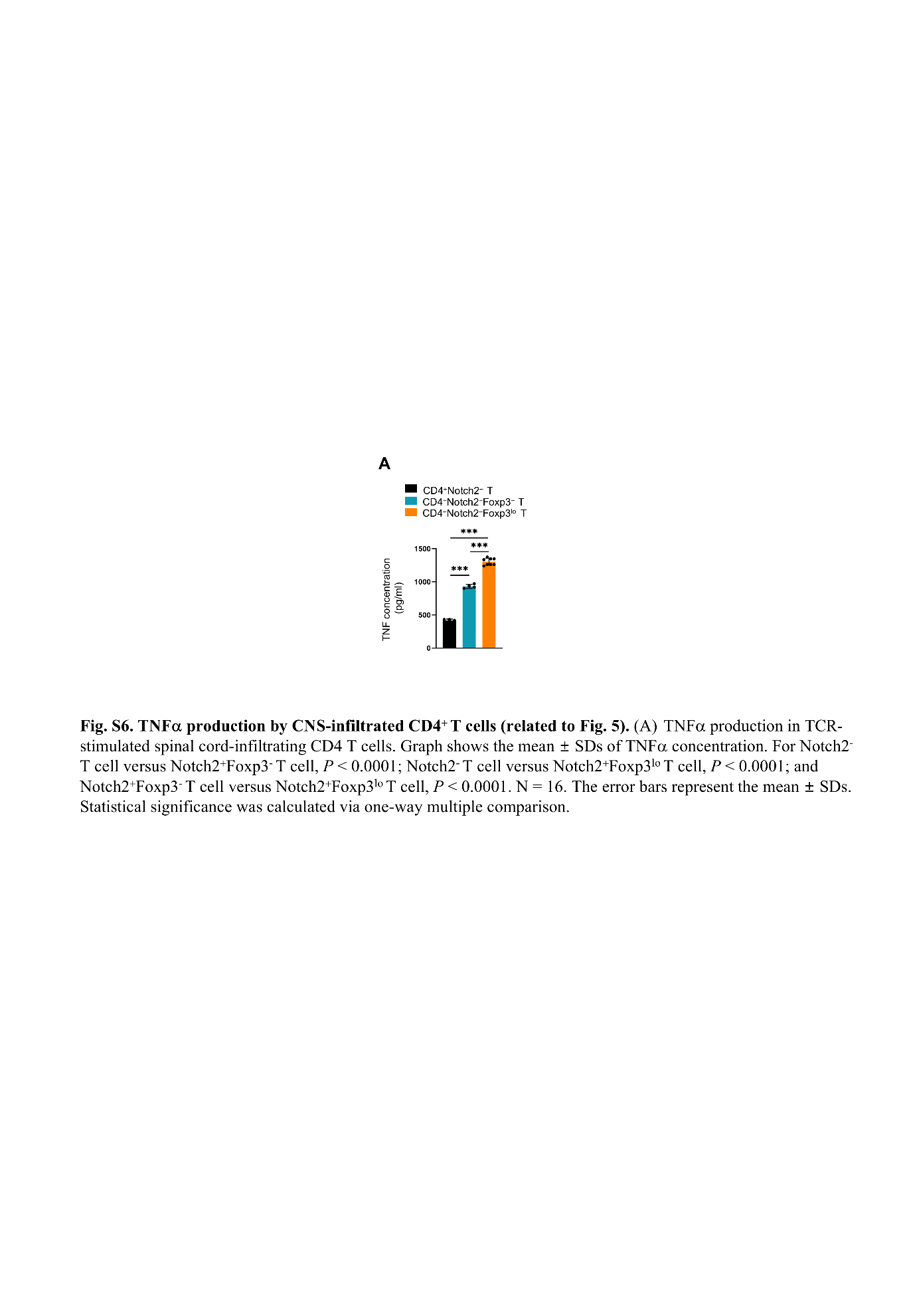
Supplementary Fig. 9. TNFα production by CNS−infiltrated CD4^+^ T cells (related to Fig. 5). A TNFα production in TCR−stimulated spinal cord−infiltrating CD4^+^ T cells. Graph shows the mean ± SDs of TNFα concentration. Data are representative of two (A) independent experiments (error bars, s.d. of >four (A) mice). Statistical significance was calculated via one−way multiple comparison; ****P* < 0.001.


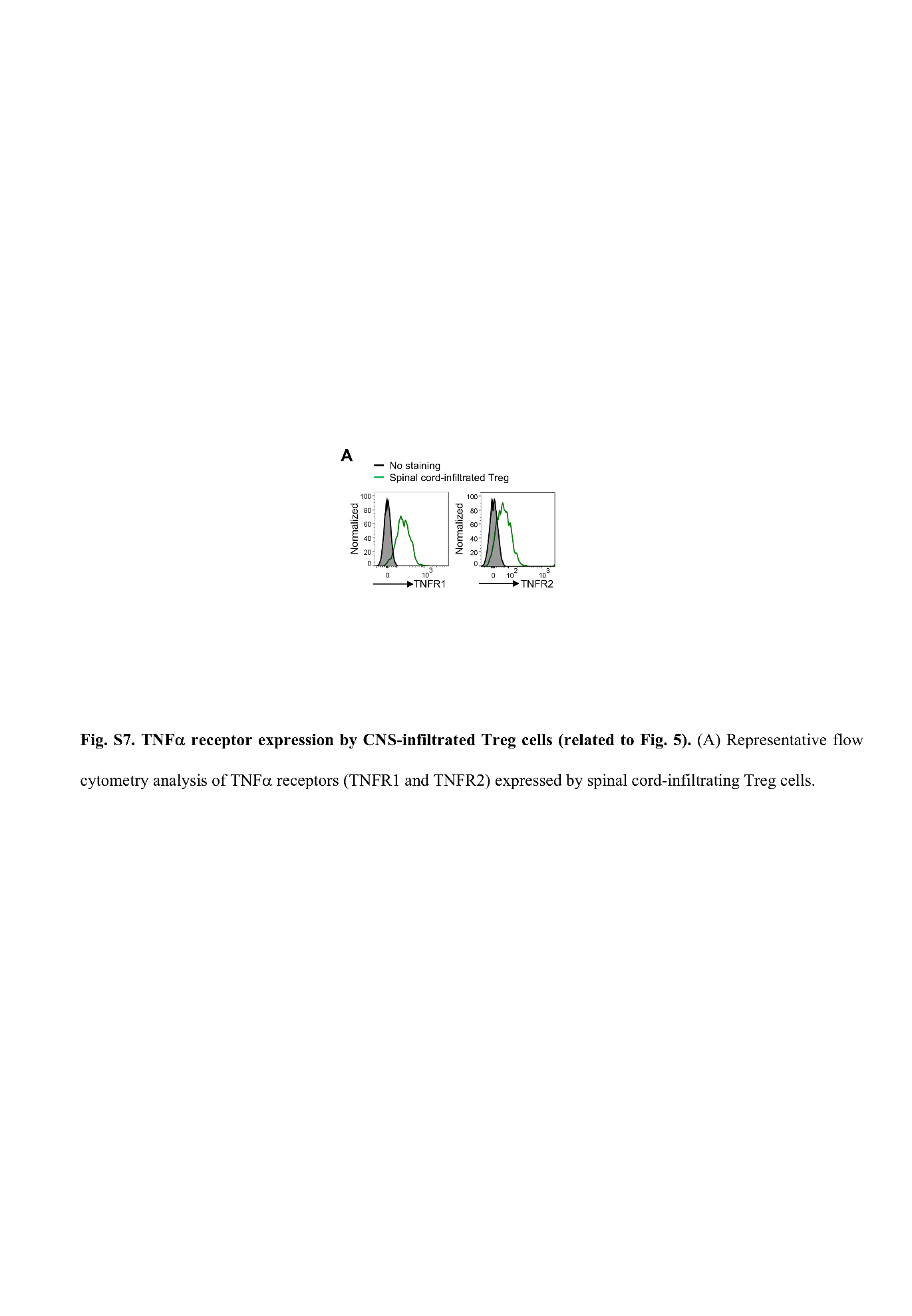
Supplementary Fig. 10. TNFα receptor expression by CNS−infiltrated Treg cells (related to Fig. 5). A Representative flow cytometry analysis of TNFα receptors (TNFR1 and TNFR2) expressed by spinal cord−infiltrating Treg cells. Data are representative of two (A) independent experiments.


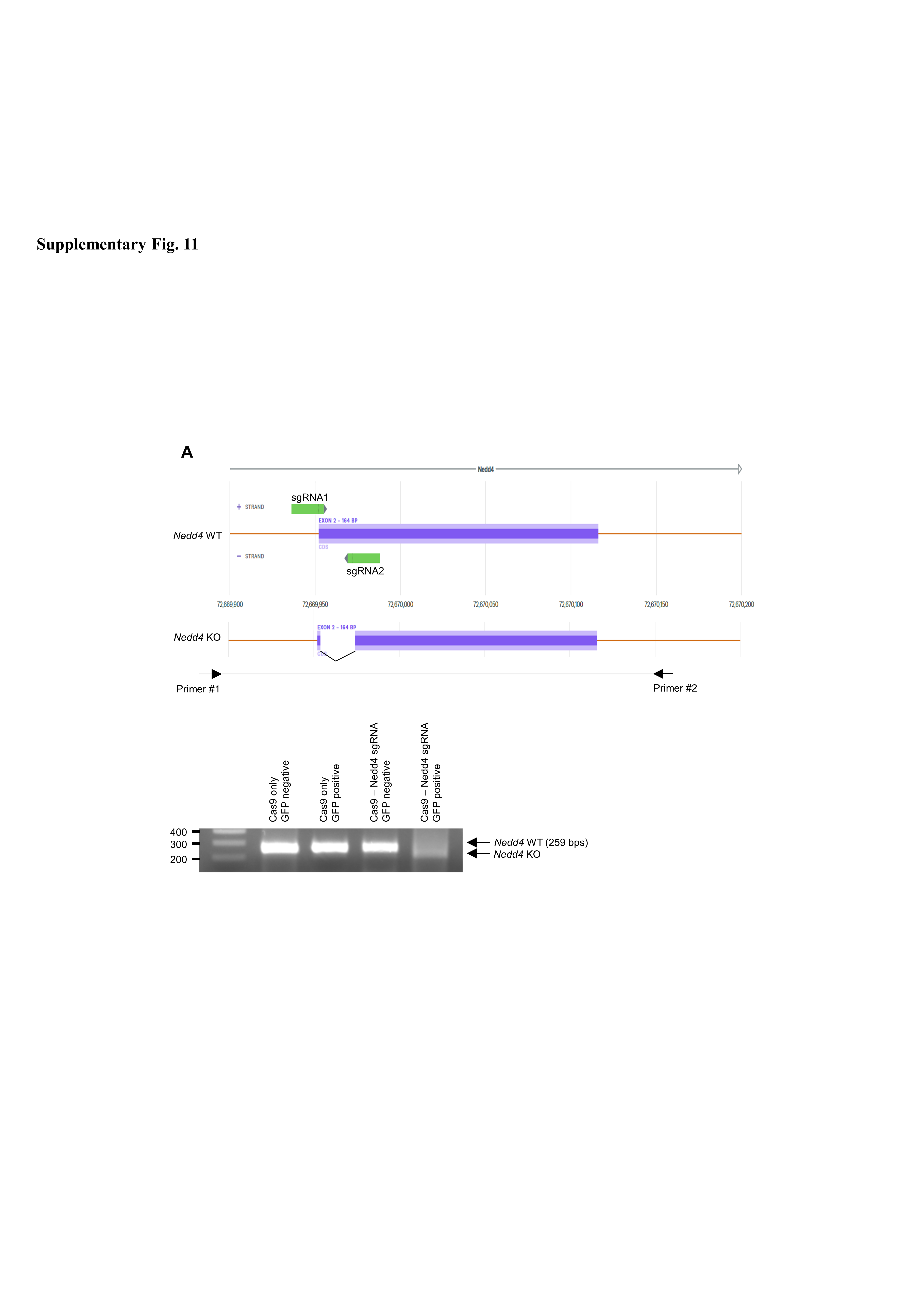


Supplementary Fig. 11. *Nedd4* deletion by CRISPR/Cas9 system (related to Fig. 6). A Schematic showing the CRISPR−mediated modification and Gel-electrophoresis image of Nedd4 fragments after CRISPR−mediated modification activity. PCR products were analyzed by 2 % agarose gel. Data are representative of two (A) independent experiments.


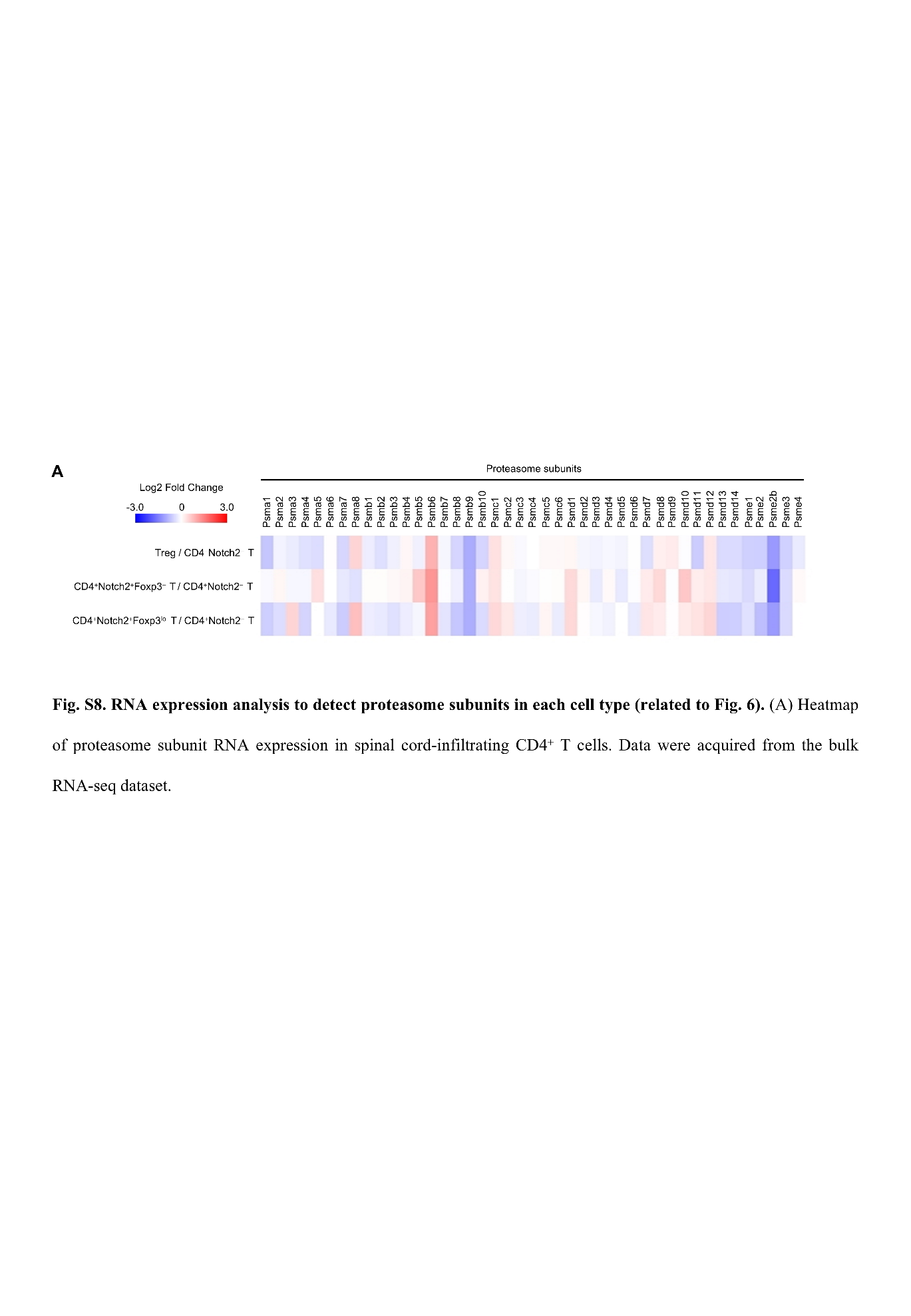
 Supplementary Fig. 12. RNA expression analysis to detect proteasome subunits in each cell type (related to Fig. 6). A Heatmap of proteasome subunit RNA expression in spinal cord−infiltrating CD4^+^ T cells. Data were acquired from the bulk RNA−seq dataset.


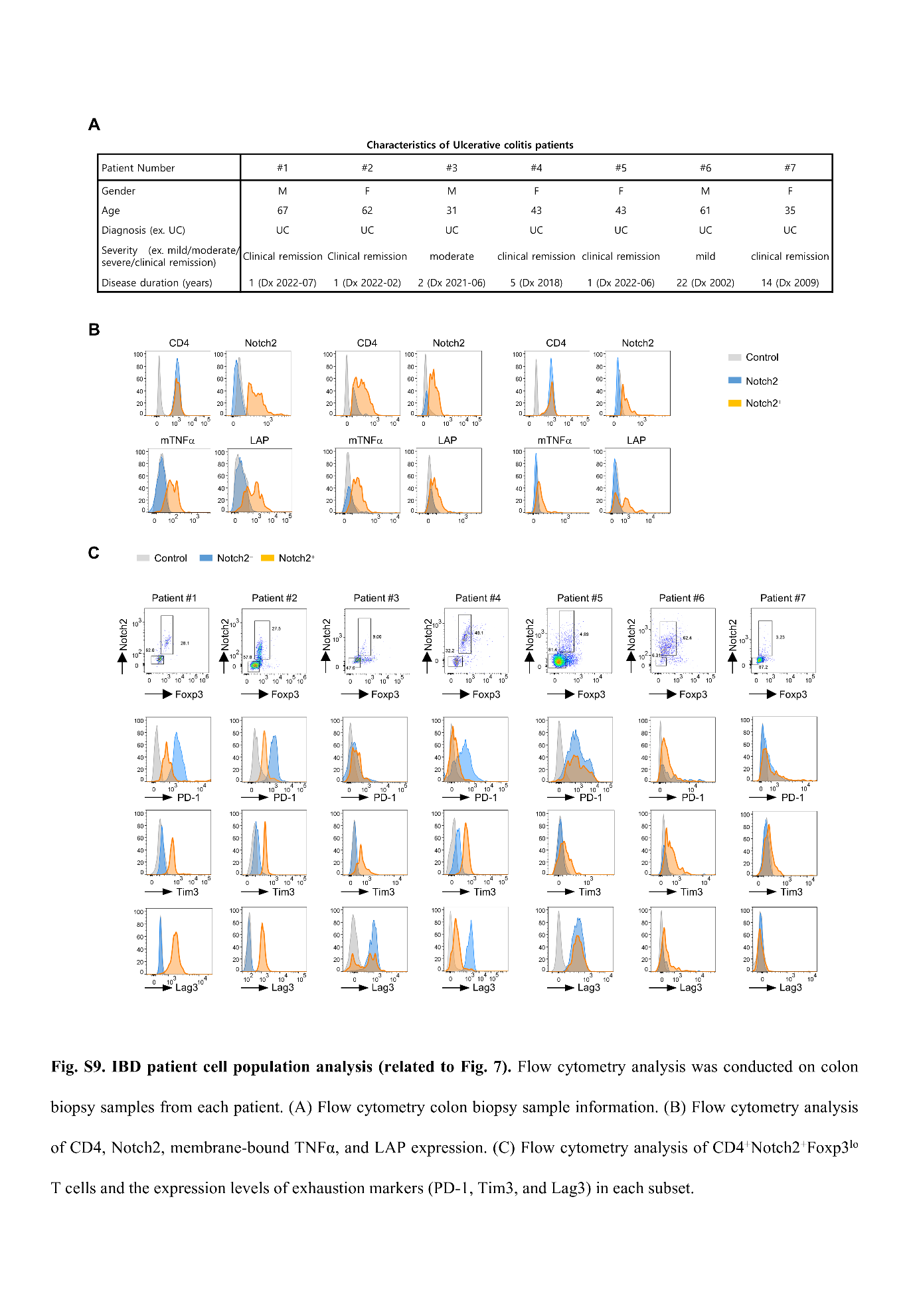


Supplementary Fig. 13. IBD patient cell population analysis (related to Fig. 7). Flow cytometry analysis was conducted on colon biopsy samples from each patient. (A) Flow cytometry colon biopsy sample information. (B) Flow cytometry analysis of CD4, Notch2, membrane-bound TNFα and LAP expression. (C) Flow cytometry analysis of CD4^+^Notch2^+^Foxp3^lo^ T cells and the expression levels of exhaustion markers (PD−1, Tim3, and Lag3) in each subset.

| Oligonucleotides | | |
| --- | --- | --- |
| Mouse *Hes5* primer: (F) CCG TCA GCT ACC TGA AAC ACA G | Macrogen | N/A |
| Mouse *Hes5* primer: (R) GGT CAG GAA CTG TAC CGC CTC | Macrogen | N/A |
| Mouse *DLL1* primer: (F) GGG CTT CTC TGG CTT CAA CT | Macrogen | N/A |
| Mouse *DLL1* primer: (R) CAC TTG GCA CCG TTA GAA CA | Macrogen | N/A |
| Mouse *DLL3* primer: (F) TAT GCA CGA GAG GCC TGA TC | Macrogen | N/A |
| Mouse *DLL3* primer: (R) TCA GCC TGA TGT GGT TGA GC | Macrogen | N/A |
| Mouse *DLL4* primer: (F) AGG TGC CAC TTC GGT TAC ACA G | Macrogen | N/A |
| Mouse *DLL4* primer: (R) CAA TCA CAC ACT CGT TCC TCT CTT C | Macrogen | N/A |
| Mouse *Jag1* primer: (F) CCT CGG GTC AGT TTG AGC TG | Macrogen | N/A |
| Mouse *Jag1* primer: (R) CCT TGA GGC ACA CTT TGA AGT A | Macrogen | N/A |
| Mouse *Jag2* primer: (F) TTC CAC AGG TCT GTT GGT GC | Macrogen | N/A |
| Mouse *Jag2* primer: (R) AGA GTA TGT CCT TGT GGC CC | Macrogen | N/A |
| Mouse *Gapdh* primer: (F) GCC TTC TCC ATG GTG GTG AA | Macrogen | N/A |
| Mouse *Gapdh* primer: (R) GCA CAG TCA AGG CCG AGA AT | Macrogen | N/A |
| Mouse *Notch2* primer: (F) CAG CCT T A AGC ACA GAC AG | Macrogen | N/A |
| Mouse *Notch2* primer: (R) GGC ATC CA ATC GGT TAC AC | Macrogen | N/A |
| Mouse *Nedd4* primer: (F) TCG GAG GAC GAG GTA TGG G | Macrogen | N/A |
| Mouse *Nedd4* primer: (R) GGT ACG GAT CAG CAG TGA ACA | Macrogen | N/A |

Supplementary Table 1.

List of oligonucleotides used in the study.

Supplementary Video 1. (separate file) CD4^+^Notch2^+^Foxp3^lo^ T cells interact directly with Treg cells (related to Fig. 5)
