# Supplementary figures and images for: Notch2−expressing CD4+ T cells attain immunoregulatory functions during autoimmune inflammation
Source: Cell Mol Immunol. 2025 Jul 23;22(9):1077–92. doi: 10.1038/s41423-025-01318-2 (PMC12398495; doi:10.1038/s41423-025-01318-2)

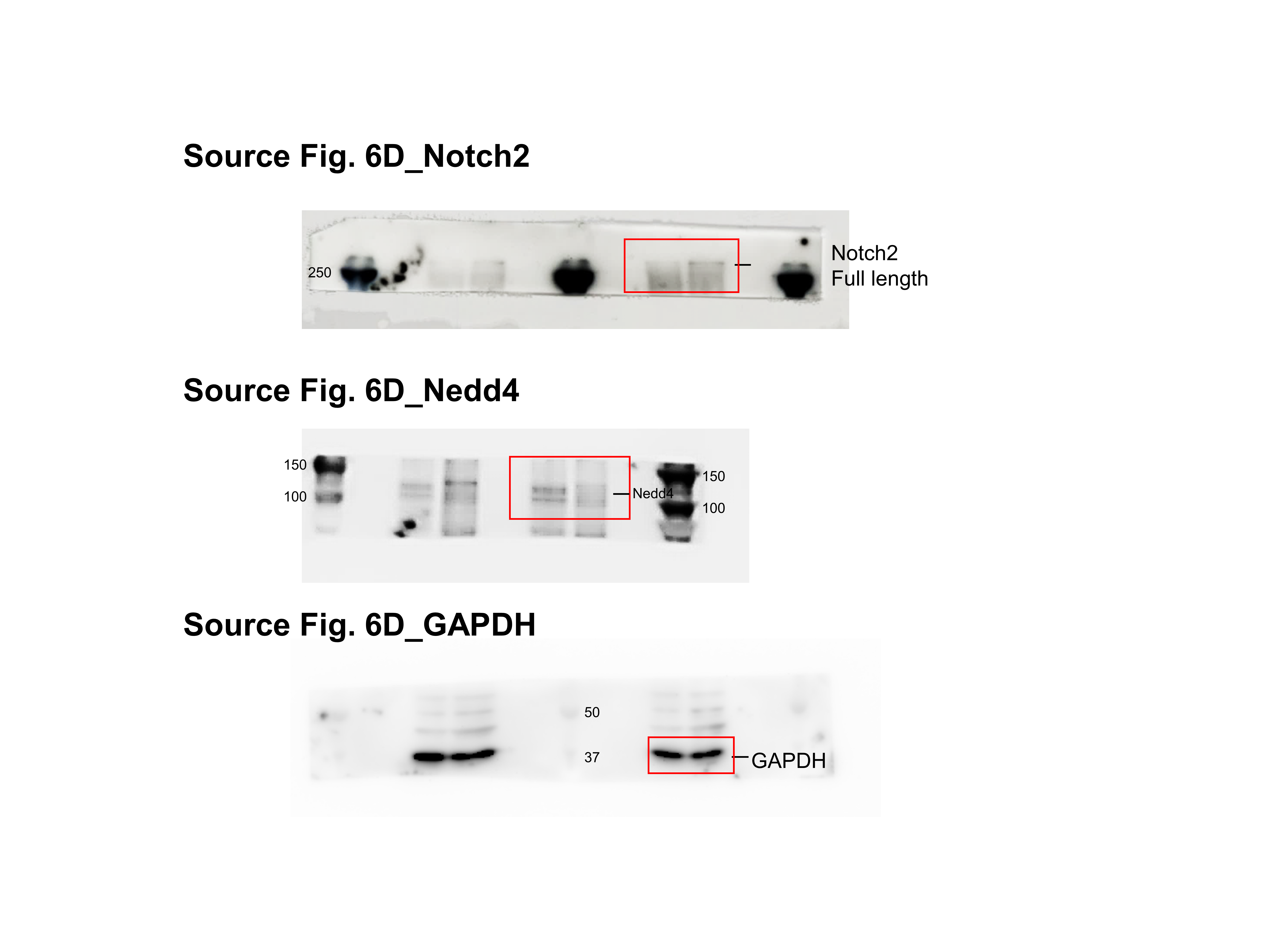

Supplement: Supplementary file 3 — Source for Fig. 6D [file 41423_2025_1318_MOESM3_ESM.tif]

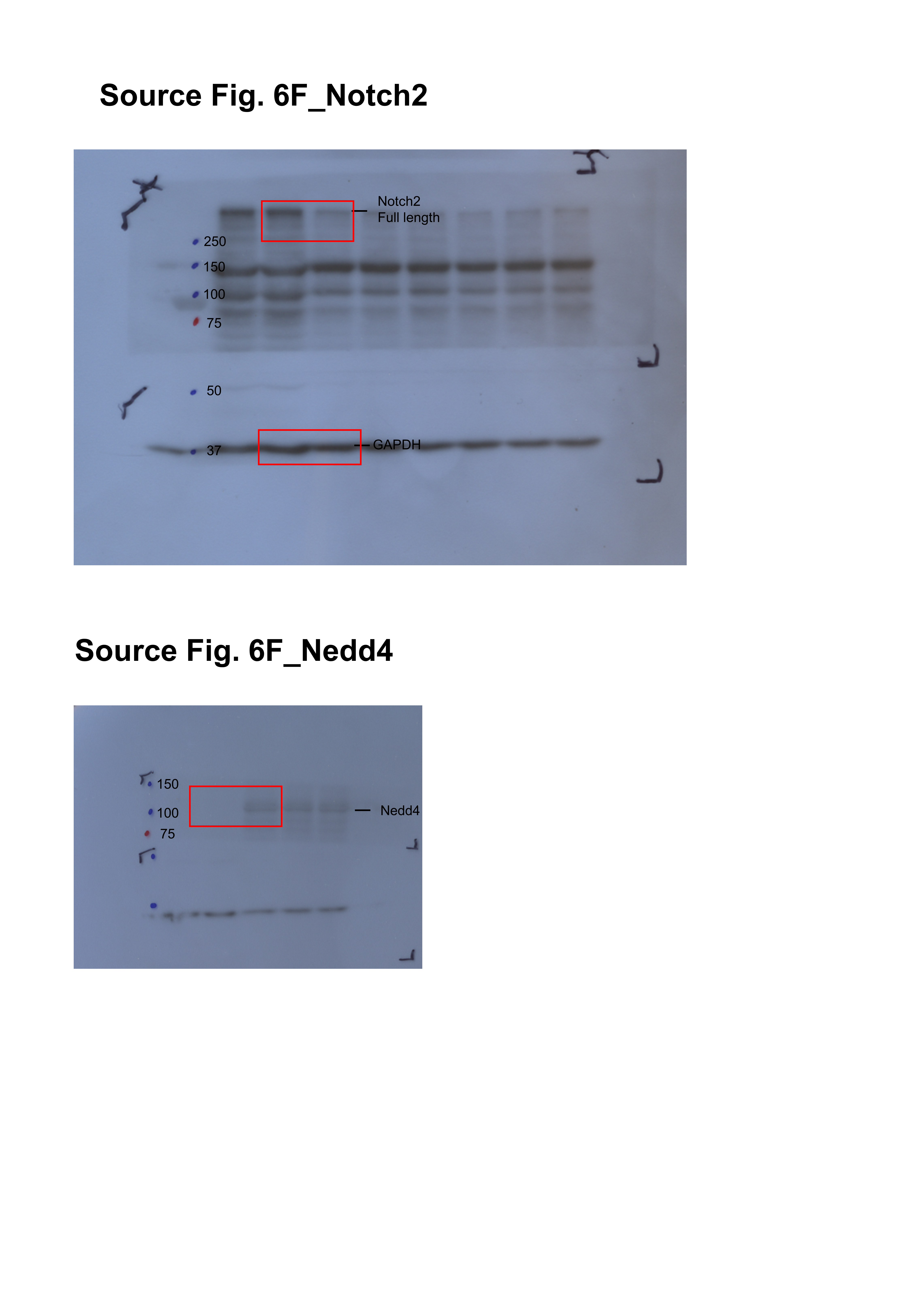

Supplement: Supplementary file 4 — Source for Fig. 6G [file 41423_2025_1318_MOESM4_ESM.tif]
